# Metal Preference Hierarchy in the HDAC8 Active Site: A DFT Study

**DOI:** 10.3390/molecules31020306

**Published:** 2026-01-15

**Authors:** Nikolay Toshev, Diana Cheshmedzhieva, Yordanka Uzunova, Kristiyan Velichkov, Todor Dudev

**Affiliations:** 1 Department of Bioorganic Chemistry, Faculty of Pharmacy, Medical University of Plovdiv, 15A Vassil Aprilov Blvd, 4002 Plovdiv, Bulgaria; 2 Department of Pharmaceutical and Applied Organic Chemistry, Faculty of Chemistry and Pharmacy, Sofia University “St. Kliment Ohridski”, 1 James Bourchier Blvd, 1164 Sofia, Bulgaria; dvalentinova@chem.uni-sofia.bg (D.C.); t.dudev@chem.uni-sofia.bg (T.D.); 3 Research Institute, Medical University of Plovdiv, 15A Vassil Aprilov Blvd, 4002 Plovdiv, Bulgaria; 4 Medical Faculty, Medical University of Plovdiv, 15A Vassil Aprilov Blvd, 4002 Plovdiv, Bulgaria; kristiyan.velichkov4@gmail.com

**Keywords:** DFT study, HDAC8 active site, metal ion selectivity, metal competition, HDACi

## Abstract

HDAC8 is a histone deacetylase enzyme that plays a key role in the development of various diseases in humans, including cancers, neurodegenerative diseases, and alcohol use disorder. Although HDAC8 is classified as a Zn^2+^-dependent metalloenzyme, available data regarding the affinity of other biologically relevant ions, such as Fe^2+^, Ni^2+^, Co^2+^, and Mg^2+^, for the HDAC8 enzyme active site remain unclear and contradictory. The mechanism by which these ions compete with Zn^2+^ for the HDAC8 active site is not well understood. In this study, we performed density functional theory (DFT) calculations at the B3LYP/6-31+G(d) level of theory, combined with polarizable continuum model computations (PCM) in water (ε = 78) and methanol (ε = 32). The results show that Zn^2+^ remains the thermodynamically preferred cofactor across all modeled reactions. Although Fe^2+^ and Co^2+^ gain partial stabilization upon increasing coordination number, the associated entropic and desolvation penalties prevent them from outcompeting Zn^2+^ under physiologically relevant conditions. Only a limited number of substitution reactions for Fe^2+^ and Co^2+^ yield ∆G values near thermodynamic neutrality, and only in specific coordination states. In contrast, all modeled Ni^2+^ substitution reactions are unfavorable, and Mg^2+^ is strongly excluded from the HDAC8 active site in all reactions. The resulting metal preference hierarchy—Zn^2+^ > Co^2+^ ≈ Fe^2+^ > Ni^2+^ > Mg^2+^—supports experimental observations and clarifies the intrinsic selectivity of the HDAC8 enzyme towards Zn^2+^. These insights provide a molecular basis for understanding HDAC8 metallo-regulation and may guide the rational design of novel, isoform-specific HDACi with improved binding properties.

## 1. Introduction

### 1.1. Cancer, Histones, and Histone Modification

Chromatin structure and histone modifications are essential in the regulation of gene expression. Histone proteins are exposed to different covalent modifications, such as acetylation, phosphorylation, and sumoylation, called post-translational modifications [1]. Acetylation of lysine residues on histone tails neutralizes their positive charge, leading to a relaxed chromatin structure.

### 1.2. HDACs and Their Biological Significance

Histone deacetylases (HDACs) are a group of enzymes that catalyze the removal of acetyl residues from acetylated histones and non-histone proteins, producing lysine and acetate. This post-translational modification (PTM) of the chromatin structure plays a crucial role in different biological processes, and it is related to various diseases, such as cancers, neurodegenerative diseases, parasitic diseases, and alcohol use disorder [2,3,4]. Among various PTMs, histone acetylation has attracted scientific interest due to its role in chromatin remodeling and regulation of gene expression [5].

The acetylation status of histones is controlled by two opposite classes of enzymes, which have antagonistic functions—HATs (histone acetyl transferases) and HDACs (histone deacetylases). Aberrant activity of these enzymes is observed in different types of cancers, and it is related to the stage of disease development and prognosis [2]. This disruption is caused by the abnormal activity of HATs, involved in the process of histone acetylation, while HDACs are responsible for the histone deacetylation [1]. Thus, HDACs have emerged as important drug targets [6], and therapies aim to restore the disrupted acetylation–deacetylation balance. Nowadays, epigenetic therapy is considered one of the most promising approaches for the treatment of many diseases, such as cancer, neurodegenerative diseases, diabetes, alcohol use disorder, and so on.

### 1.3. Classification of HDACs

The human HDACs are a class of enzymes, consisting of 18 human enzymes, which are grouped in four classes. Classes I, II, and IV are considered as metalloenzymes, while class III requires NAD+ as a cofactor [7]. Among class I enzymes, HDAC8 (containing 377 amino acids) is found in both the nucleus and cytoplasm [8]. HDAC8 is the most important and well-studied of all metalloproteins [8,9]. Its role in cancer is a subject of intensive study due to the key role of this metalloprotein in different cancers such as T-cell lymphoma, childhood neuroblastoma, gastric cancer, colon cancer, breast cancer, and acute myeloid leukemia [10]. Drug therapy relies on inhibiting the enzymatic activity of HDAC8 through the use of small inhibitor molecules called inhibitors of histone deacetylase HDACi [11,12].

### 1.4. HDAC Inhibitors: Classification and Approved Drugs

**Figure 1:**
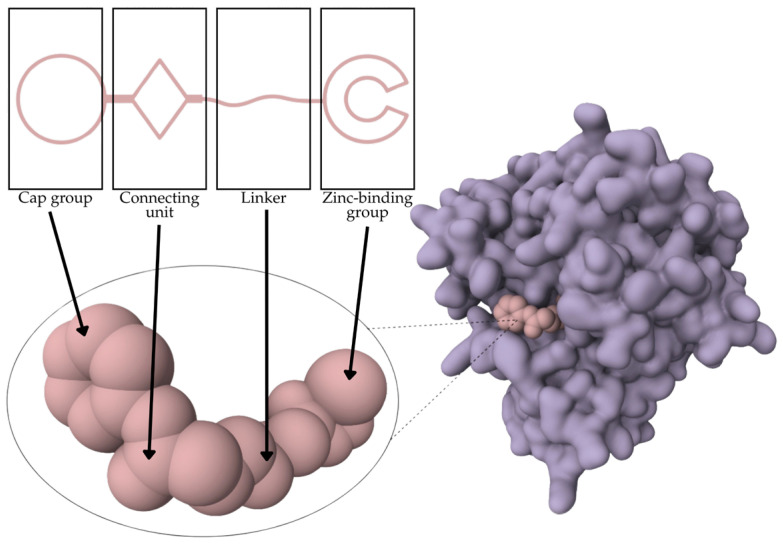
Pharmacophore model of HDACis.

Various HDAC inhibitors have been developed and categorized into different chemical classes [13,14]. Key examples include hydroxamic acids (e.g., suberoylanilide hydroxamic acid, SAHA or Vorinostat [15], Belinostat [16], and Panobinostat [17]); benzamides, such as Chidamide [18]; cyclic tetrapeptides (Romidepsin, FK 228 [19,20]); short-chain fatty acids; and others [13]. Despite their structural diversity [21], they share a common pharmacophore model: they comprise the following subunits—cap group, carbon linker, connecting unit, and metal-binding group head [13,22] (Figure 1). In the HDACs active site, the hydroxamic moiety, commonly known as the hydroxamic head, chelates the catalytic metal ions, thus exhibiting its inhibitory activity over the enzyme [13,23,24]. From a chemical standpoint, HDACi can be classified in the following chemical classes: hydroxamic acids, benzamides, short-chain fatty acids, and cyclic tetrapeptides (Table 1).

### 1.5. Pan-HDAC vs. Isoform-Specific HDAC Inhibition

Current HDAC inhibitors inhibit multiple HDAC isoforms, classifying them as pan-HDACi [25]. However, their broad activity is associated with adverse effects in patients. Effects such as fatigue, thrombocytopenia, and cardiac effects are believed to result from the lack of isoform specificity in these first-generation HDACis [26]. Consequently, there is a need to develop isoform-specific inhibitors [27], thus reducing side effects and improving therapeutic index.

### 1.6. HDAC8—Metal Dependence, Selectivity, and Competition

The identity of the metal cofactor in metalloenzymes remains one of the intriguing questions in elucidating metalloprotein structure, function, and regulation [28]. Histone deacetylase 8 (HDAC8) is a class I HDAC metalloenzyme whose catalytic activity depends on the nature of the metal ion within its active site. The native Zn^2+^ is pentacoordinated, ligated by a hydroxamate group (in an inhibitor-bound molecule), by water, or by three amino acid residues (Asp178, Asp267, and His180) [29].

Although HDAC8 is classified as a Zn^2+^-dependent metalloenzyme, previous studies have demonstrated that several biogenic divalent ions, including Fe^2+^, Co^2+^, and Ni^2+^, can also bind to the active site and support varying levels of catalytic activity. In the cellular environment, different divalent cations, in particular Zn^2+^, Mg^2+^, and Fe^2+^, exist in concentrations sufficient to compete for metal-binding sites in enzymes. This competition has inspired scientific interest in elucidating the factors that influence metal affinity and selectivity, such as metal-ion size, ligand-field preferences, coordination geometry, and intracellular availability [30]. Biochemical studies have shown that HDAC8 binds various divalent metals, with its catalytic activity significantly influenced by the identity of the bound ion. Gantt and coworkers [31] reported that HDAC8 exhibits measurable activity with Co^2+^, Fe^2+^, Zn^2+^, and Ni^2+^, with decreasing catalytic efficiency in the order: Co > Fe > Zn > Ni. This data suggests that Zn^2+^ may not be the exclusive cofactor and that the HDAC8 enzyme may function under conditions where the metal substitution occurs, leading to a catalytic modulation. Structural and mechanical studies have suggested that the HDAC8 active site is accommodating towards multiple metal ions, whose coordination geometries and ligand-field stabilization differ [31,32].

Based on the Irving-William series [33], Zn^2+^ is predicted to form more stable complexes than Fe^2+^ and Mg^2+^ with different ligands [34]. However, Zn^2+^ is present in a lower concentration (10–400 pM) [35,36] compared to Fe^2+^ (0.2–0.6 µM [37,38,39]) and Mg^2+^ (mM range [40]) within the cellular environment [32]. Another important factor is the similarity in ionic radii (r(Fe^2+^) = 0.78 Å, r(Mg^2+^) = 0.72 Å, and r(Zn^2+^) = 0.74 Å) when they are hexacoordinated [28,41], and they have similar coordination ability, forming hexacoordinated complexes. In addition to these biologically abundant cations, first-row transition metals such as Co^2+^ and Ni^2+^ also require attention. They demonstrate significant ligand-field stabilization energies, forming thermodynamically stable complexes, often comparable to or exceeding those of Fe^2+^ [42]. In contrast, the biological significance of Co^2+^ and Ni^2+^ is limited by their extremely low intracellular free concentrations [43]. Their ionic radii r(Co^2+^) ≈ 0.74 Å and r(Ni^2+^) ≈ 0.69 Å [44] are like those of Zn^2+^, suggesting structural compatibility with different metalloprotein active sites. However, their intracellular concentrations are extremely low (in the pm–nm range), minimizing their physiological significance despite their advantageous coordination chemistry. Free Co^2+^/Co^3+^ levels are in the picomolar–nanomolar range, as a cofactor in cobalamin. The concentrations of free Ni^2+^ are even lower (in the pm range), as mammalian cells have only a small number of Ni-dependent enzymes and keep strict regulatory control due to the metal’s potential toxicity [43]. Thus, despite exhibiting strong thermodynamic complexation tendencies, Co^2+^ and Ni^2+^ are unlikely to compete with Zn^2+^ for HDAC8 binding under physiological conditions. These observations, together with the structural similarities of the respective metal complexes, raise fundamental questions regarding the determinants of metal selectivity in HDAC8.

### 1.7. Research Gap and Objectives of the Study

Despite increasing experimental insights regarding the ability of HDAC8 to accommodate multiple metal ions, the thermodynamic principles governing the competition between Zn^2+^ and biologically relevant divalent cations (e.g., Fe^2+^, Mg^2+^, Co^2+^, and Ni^2+^) remain unclear. A comprehensive understanding of metal identity and binding characteristics is essential for the design of new isoform-specific HDACi. Although a substantial body of information is available on HDACs and HDACi, a number of outstanding questions remain unanswered: 1. What is the order of HDAC8’s affinity/selectivity for such biogenic divalent ions, such as Fe^2+^, Mg^2+^, Co^2+^, and Ni^2+^, which are present in cellular fluids, and is it possible to activate the HDAC8 enzyme by other ions present in the cellular fluids, thus interfering with the HDAC8 enzyme function? 2. Is it possible for other divalent biogenic ions (Fe^2+^, Mg^2+^, Co^2+^, and Ni^2+^), considered as “non-native,” which are present in the cellular environment, to outcompete Zn^2+^ for binding the HDAC8 active site? 3. What are the governing factors influencing metal ion competition and selectivity in the HDAC8 enzyme?

In this context, the term “metal hierarchy” refers to the relative thermodynamic favorability of different biogenic divalent metal ions for binding the HDAC8 active site. This ranking is based on the computed Gibbs free energies of metal-substitution reactions using Zn^2+^ as a reference.

## 2. Results and Discussion

The identity and coordination behavior of the metal cofactor in HDAC8 directly affect inhibitor binding geometry due to chelation of the active site metal ion. Therefore, understanding HDAC8’s thermodynamic preference for specific divalent biogenic metal ions provides a necessary framework for elucidating enzyme–inhibitor interactions and the metal-dependent inhibition of HDAC8.

To assess the thermodynamic competitiveness of biologically relevant metal ions (M^2+^ = Fe^2+^, Ni^2+^, Mg^2+^, and Co^2+^) in the HDAC8 enzyme site, a systematic series of substitution reactions was modeled. Free energies (∆G) were calculated for each exchange across all coordination states (L4, L5, and L6), both in the gas phase and in polar solvents mimicking physiological conditions, using Zn-bound complexes as the reference. The results reported here allow for developing a hierarchy of metal preferences for the HDAC8 active site under physiological-like conditions.

Fe^2+^ → Zn^2+^ exchange

The calculated free energies for substituting Zn^2+^ with Fe^2+^ in HDAC8 (Table 2) are mostly positive in aqueous solutions, indicating a strong preference for Zn^2+^. In the gas phase, only one reaction pathway (L4 → L6) gives a slightly favorable ∆G (−1.77 kcal/mol). All other reaction pathways result in positive free energy changes, with ∆G^78^ ranging from 6 to 17 kcal/mol, confirming that Fe^2+^ substitution is disfavored in polar media.

From the hard and soft acids and bases (HSAB) [45] perspective, Fe^2+^ is classified as a “hard” acid due to its small ionic radius and high charge density, whereas Zn^2+^ is considered a borderline acid. On one side, Fe^2+^ has a strong affinity to hard, oxygen-donor ligands; on the other hand, the HDAC8 enzyme site includes histidine residues that incorporate softer nitrogen atoms and display more favorable interaction with Zn^2+^. Additionally, Zn^2+^ does not have ligand field stabilization, allowing it to assume flexible four- or five-coordinate geometries, whereas Fe^2+^ favors an octahedral configuration.

Reactions that increase Fe^2+^ coordination, such as L4 → L6 and L5 → L6, show a favorable change in electronic energies (∆E = −23.33 and −12.70 kcal/mol, respectively). However, the corresponding Gibbs energies increase significantly with increasing the dielectric constant, indicating that entropic and solution effects outweigh the electronic stabilization. The only slightly favorable ∆G values occur in the gas phase (∆G^1^ = −1.77 and −0.62 kcal/mol), whereas all the corresponding values in methanol (∆G^32^) and water (∆G^78^) are positive, implying that the Zn^2+^ substitution is disfavored in polar environments. Furthermore, reactions that start from a fully solvated Zn^2+^ complex [Zn-L_6_] are unfavorable (∆G^1^ > 8.40 kcal/mol), highlighting the importance of starting geometry in the substitution process.

In conclusion, while Fe^2+^ can approach thermodynamic neutrality only in low-dielectric conditions and specific states, Zn^2+^ remains a thermodynamically favored ion across all dielectric media and coordination states.

Co^2+^ → Zn^2+^ exchange

The behavior of Co^2+^ in substitution reactions (Table 3) is similar to that of Fe^2+^ (Table 2). While most reactions are mildly endergonic, there are narrow “windows” where Co^2+^ almost competes with Zn^2+^. Notably, replacing Zn^2+^ in L5 or L6 coordination modes with Co^2+,^ forming a five-coordinate Co^2+^ complex [Co-L_5_], yields ΔG values near zero or slightly negative in polar solvent (ΔG^78^ ≈ −1.8 and −0.3 kcal/mol in two different reaction pathways). These examples indicate that solvation in high dielectric media can tip the balance in favor of Co^2+^, particularly in specific coordination environments. Nonetheless, most Co^2+^ substitutions remain slightly uphill (ΔG^78^ ≈ +5 to +15 kcal/mol), because the addition of ligands still costs an entropy/solvation penalty even if ΔE^1^ is favorable (L4 → L6: ΔE^1^ = −18.87 kcal/mol, but ΔG^78^ = +15.69 kcal/mol).

From an HSAB perspective, Co^2+^ (d^7^, high spin) is also a borderline “hard” acid similar to Fe^2+^ and can bind both oxygen and nitrogen donors [45]. The computed results show that Co^2+^ can utilize the same nitrogen/oxygen ligand set as Zn^2+^ and even achieve exchange when forming five-coordinate species, but it still pays a penalty for each additional ligand relative to Zn^2+^’s flexibility. Due to its filled d-shell, the Zn^2+^ ion has no ligand-field strain and can easily accommodate different numbers of ligands [46].

Moreover, the Irving–Williams series predicts that Zn^2+^ complexes are intrinsically more stable than Co^2+^ complexes with the same set of ligands [33]. Thus, although Co^2+^ occasionally approaches Zn^2+^ energetically in polar media, Zn^2+^ retains the overall preferred ion.

The Co^2+^ → Zn^2+^ substitution free energies in HDAC8 complexes remain positive, indicating that Zn^2+^ is preferred over Co^2+^. However, ∆G values for Co^2+^ substitution are generally lower than those observed for Mg^2+^ or Ni^2+^ (see below) and in some coordination environments are comparable to or only slightly greater than those for Fe^2+^. This behavior suggests that Co^2+^ has a limited ability to compete with Zn^2+^ in the HDAC8 active site. It is more competitive than Mg^2+^ and is almost as competitive as Fe^2+^, but it is not universally favored. Most substitution reactions yield moderately positive ∆G values. Similarly to the Fe^2+^ vs. Zn^2+^ exchange, two exchange reactions [Zn–L_5_] → [Co–L_5_] and [Zn–L_6_] → [Co–L_5_] + W, become favorable in polar media, supporting the conclusion that solvation helps Co^2+^ to compete in specific coordination states. Despite this, Zn^2+^ remains the ion of choice and has an overall advantage.

Mg^2+^ → Zn^2+^ exchange

The Gibbs free energies for Mg^2+^ → Zn^2+^ substitution in the modeled HDAC8 enzyme site (Table 4) are strongly positive across all coordination states and reaction pathways, indicating that all modeled exchanges are thermodynamically unfavorable. Both ∆E and ∆G values are consistently endergonic and positive, with ∆G exceeding +50 kcal/mol in several cases. Compared to the previous metal ion exchange, no borderline or favorable cases are observed. Mg^2+^ never approaches thermodynamic neutrality; every coordination environment predicts exclusion of Mg^2+^ from the HDAC8 site. This behavior can be explained by the chemical nature of magnesium: it is a “hard acid,” characterized by a small ionic radius and increased charge density. It has an octahedral geometry in solution, coordinated by six water molecules, and demonstrates a strong preference for oxygen-containing molecules. As a result, the magnesium ion is too “hard” and strongly hydrated to integrate into the HDAC8 model site. In contrast, the HDAC8 active site requires some degree of coordination flexibility and accommodates mixed nitrogen-oxygen ligand environments. The enzyme’s moderate flexibility and mixed N/O ligand structure cannot overcome magnesium’s rigid octahedral preference and strong hydration shell. Despite its high intracellular abundance, Mg^2+^ is not observed as a cofactor for HDAC8.

Ni^2+^ → Zn^2+^ exchange

The Ni^2+^ → Zn^2+^ substitution free energies (Table 5) remain positive, indicating that the substitution is thermodynamically unfavorable in the apo-HDAC8 enzyme active site. Although several reaction pathways involving increased coordination of Ni^2+^ exhibit favorable electronic energies (∆E = −12.70 and −5.67 kcal/mol), the corresponding ∆G values remain positive. Like Co^2+^ and Fe^2+^, Ni^2+^ exhibits electronic stabilization when its coordination number increases (L4 → L6 gives ∆E = −23.33 kcal/mol). On the other hand, when considering changes in Gibbs free energy, all computed Ni^2+^ → Zn^2+^ substitution reactions are endergonic, with even the best pathways resulting in ∆G^78^ values equaling approximately +3 to +4 kcal/mol. This suggests that entropy and solvation effects outweigh the electronic stabilization.

From an HSAB point of view, Ni^2+^ is a borderline Lewis acid with a d^8^ electronic configuration, exhibiting strong ligand-field stabilization in an octahedral geometry. It strongly favors six-coordinated environments with rigid ligands. Additionally, Ni^2+^ is nearly as strongly hydrated as Co^2+^ and Fe^2+^, so replacing its hydration shell with biological ligands significantly increases desolvation costs. Compared to Co^2+^, which showed borderline substitution in some pathways, Ni^2+^ never achieves a favorable exchange. This supports previous data about stability trends: Ni^2+^ complexes are less stable than Zn^2+^ complexes, and their affinity for octahedral coordination makes them unsuitable for the flexible HDAC8 environment. Moreover, these results confirm that Ni^2+^ ions cannot compete with Zn^2+^ ions for binding in the HDAC8 enzyme site under any of the examined conditions.

Additionally, in an attempt to support the observed thermodynamic trends, we examined the metal–ligand bond lengths in the optimized structures, which sheds more light on the relative binding ability of the studied metal ions within the HDAC8 active site.

The bond length analysis aligns with the coordination patterns across the metal ions in the HDAC8 enzyme active site. The Zn–N (His180) bond length varies from 2.00 to 2.04 Å, consistent with known structural data for zinc coordination in metalloenzymes [22]. Fe^2+^ and Co^2+^ show comparable distances to His180, ranging from 2.09 to 2.12 Å and 2.01–2.05 Å, respectively, while Mg^2+^ demonstrates slightly longer distances (2.13–2.14 Ǻ) and Ni^2+^ (2.00–2.01 Å). For oxygen donors, Zn–O bond lengths to Asp178 and Asp267 fall within 1.98–2.01 Å and 1.97–1.98 Å, respectively, indicating strong binding. Similarly, for Fe^2+^ and Co^2+^ ranges observed were 1.97–2.03 Å. The slightly longer Asp178 and Asp267 M-O distances for Ni^2+^ and Mg^2+^ (2.02–2.12 Å) and (1.98–2.03 Å) suggest weaker coordination ability. These trends support the thermodynamic metal preference hierarchy calculated in this study. The observed bond length trends were consistent across both water and methanol.

## 3. Materials and Methods

### 3.1. Modeled Metal–Ligand Complexes

To accurately model the metal–ligand environment of the HDAC8 active site, we constructed model complexes for each divalent metal ion. It focuses on the first coordination sphere, which governs metal selectivity and complexation energetics. These complexes include two aspartate residues (Asp178 and Asp267), one histidine residue (His180), the peptide backbone of lysine (Lys), and one or two water molecules, depending on the coordination number. The notation [M-L_x_] refers to the metal–ligand environment of a metal ion M^2+^, where x = 4, 5, or 6 corresponds to tetra-, penta-, and hexacoordination, respectively. These models provide a consistent framework for comparing the binding affinities and structural preferences of the studied divalent cations within the HDAC8 active site.

Notably, the metal–amino acid interactions are mostly electrostatic in nature and rapidly fade away with distance. Thus, the contribution of ligands from the second, third, and more distant coordination layers to the overall/free energy of the system is relatively low and might be considered as a second-order effect. This study focuses on the relative substitution energies and metal preference trends; therefore, these longer-range effects are expected to cancel when comparing closely related metal–ligand complexes. This first-sphere modeling approach has been widely used in other computational studies [28,47].

**Figure 2:**
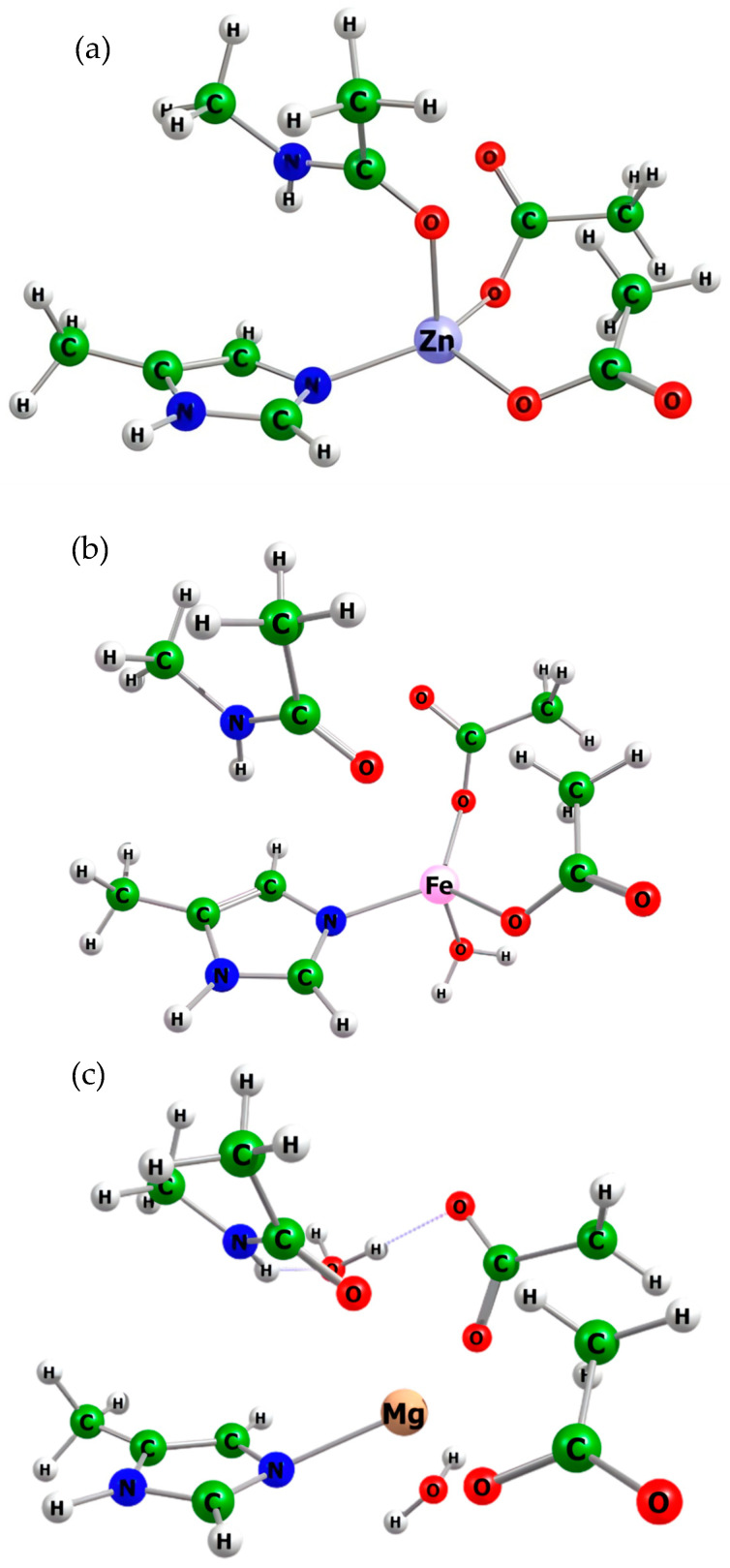
Optimized geometries of the HDAC8 active site model complexes [Zn-L_4_], [Fe-L_5_], and [Mg-L_6_] obtained at the B3LYP/6-31+G(d) level of theory. (**a**) [Zn-L_4_], characterized by a metal–ligand environment formed by Asp178, Asp267, His180, and the Lys backbone; (**b**) [Fe-L_5_], displaying a metal–ligand environment that includes one water molecule in addition to Asp178, Asp267, His180, and the Lys backbone; (**c**) [Mg-L_6_], representing a more saturated metal–ligand environment, in which two water molecules coordinate alongside Asp178, Asp267, His180, and the Lys backbone.

The structures in Figure 2 illustrate three distinct metal–ligand environments relevant to the HDAC8 catalytic center.

Zinc Complexes (Zn^2+^):

- [Zn-L_4_]: Metal–ligand environment involving Asp178, Asp267, His180, and the Lys backbone.
- [Zn-L_5_]: Metal–ligand environment involving one water molecule, Asp178, Asp267, His180, and the Lys backbone.
- [Zn-L_6_]: Metal–ligand environment involving two water molecules, Asp178, Asp267, His180, and the Lys backbone.

Magnesium Complexes (Mg^2+^):

- [Mg-L_5_]: Metal–ligand environment involving one water molecule, Asp178, Asp267, His180, and the Lys backbone.
- [Mg-L_6_]: Metal–ligand environment involving two water molecules, Asp178, Asp267, His180, and the Lys backbone.

Cobalt Complexes (Co^2+^):

- [Co-L_5_]: Metal–ligand environment involving one water molecule, Asp178, Asp267, His180, and the Lys backbone.
- [Co-L_6_]: Metal–ligand environment involving two water molecules, Asp178, Asp267, His180, and the Lys backbone.

Iron Complexes (Fe^2+^):

- [Fe-L_5_]: Metal–ligand environment involving one water molecule, Asp178, Asp267, His180, and the Lys backbone.
- [Fe-L_6_]: Metal–ligand environment involving two water molecules, Asp178, Asp267, His180, and the Lys backbone.

Nickel Complexes (Ni^2+^):

- [Ni-L_5_]: Metal–ligand environment involving one molecule of water, Asp178, Asp267, His180, and the Lys backbone.
- [Ni-L_6_]: Metal–ligand environment involving two molecules of water, Asp178, Asp267, His180, and the Lys backbone.

### 3.2. Modeling of the Substitution Reaction Scheme

To evaluate the competitive metal binding in the HDAC8 active site, we modeled a series of substitution reactions in which Zn^2+^, considered as the native ion in the HDAC8 enzyme site, is replaced by a rival biologically relevant divalent metal ion (M^2+^ = Fe^2+^, Mg^2+^, Co^2+^, and Ni^2+^).

The general equation for the reaction is

[M(H_2_O)_6_]^2+^ + nH_2_O + [Zn-L_x_] → [Zn(H_2_O)_6_]^2+^ + [M-L_x_] + mH_2_O (1)

In this reaction:

- Zinc is bound in the HDAC8 enzyme active site and then replaced by a rival divalent biogenic ion attempting to occupy the HDAC8 active site;
- [Zn-L_x_] and [M-L_x_] represent the x-coordinate complexes (x = 4, 5, or 6) of Zn^2+^ and the attacking ion M^2+^ bound to the HDAC8;
- *n* and *m* account for additional water molecules that are required to balance the equation, depending on the coordination numbers. The number of water molecules included in the metal–ligand model was chosen to complete the first coordination sphere of the metal ion in each studied metal–ligand environment. This approach ensures chemically meaningful metal coordination while allowing systematic comparison of metal substitution energetics across different metal coordination environments.

This generalized equation is complex because it accounts for all possible combinations of divalent metal ions (M^2+^ = Zn^2+^, Fe^2+^, Ni^2+^, Mg^2+^, and Co^2+^) and coordination modes, enabling evaluation of the metal substitution scenarios in the HDAC8 active site. The total number of molecules before and after the substitution reaction depends on the coordination number and specific geometry of the metal–ligand complex. This flexible reaction model accurately depicts the ligand exchange process under biological conditions and provides computed substitution-free energies for the considered reactions. This study does not aim to determine the absolute values of binding energies of the metal–ligand complexes but to establish the trends in the relative changes in these energies.

### 3.3. DFT/PCM Calculations

All calculations were performed using the Gaussian 16 program package [48]. All the structures were initially optimized in the gas phase at the B3LYP/6-31+G(d) level of theory, yielding the respective optimized structures of the studied species. Frequency calculations were subsequently carried out at the same level of theory, confirming that all optimized structures correspond to true minima on the potential energy surface, and to obtain zero-point energy and thermal corrections. No imaginary frequencies were found in the studied molecules. As a result, Gibbs free energies were calculated by combining electronic energies with the thermochemical corrections that were derived from the frequency analysis. The Cartesian coordinates of the optimized structures used in this study are available in the Supplementary Materials.

This combination of the B3LYP method [49] and the 6-31+G(d) basis set was chosen based on previous studies of hydroxamic acids [50,51] and hydroxamic acid derivatives [22,52] and our own validation in previous studies [22]. The applied computational method accurately reproduced the geometries of metal ions and enzyme complexes, demonstrating strong agreement between theoretical predictions and experimental observations.

To account for solvation effects, polarizable continuum model (PCM) calculations in methanol (ε = 32) and water (ε = 78) were conducted with all modeled species. Considering that metal-binding sites in metalloproteins are located within cavities, where the dielectric properties of the medium are more similar to the low-polarity solvents [53], the reactions were modeled in different dielectric media. Free energies in solutions were obtained by adding solvation contributions to the gas phase Gibbs free energies.

The solvation energy (∆G^ε^_solv_) was calculated as a difference between gas-phase energies and PCM energies of each complex/molecule of the respective entity: ∆G^ε^_solv_ ≈ G_el_^2^ − G_el_^1^.

In Equation (1), where the Zn^2+^ ion is bound to the HDAC8, it is replaced by a rival divalent attacking cation, M^2+^ (M = Mg^2+^, Fe^2+^, Ni^2+^, and Co^2+^). The species [Mg(H_2_O)_6_]^2+^, [Zn(H_2_O)_6_]^2+^, [Fe(H_2_O)_6_]^2+^, [Co(H_2_O)_6_]^2+^, and [Ni(H_2_O)_6_]^2+^ represent the hydrated free divalent metal species in aqueous media, whereas [Zn–L_x_] and [M–L_x_] represent the metal–ligand complexes as previously described.

To evaluate the metal affinity, the complex formed from the bound zinc cation in the HDAC8 enzyme was considered as a reference.

The gas-phase energy of metal-metal substitution is given by the following equation: ∆G^1^ = G^1^([M-L_x_]) + G^1^([Zn(H_2_O)_6_]^2+^) − G^1^([Zn-L_x_]) − G^1^([M(H_2_O)_6_]^2+^);

Additionally, for the condensed phase, we can write as follows: ∆G^x^ = ∆G^1^ + ∆G^ε^_solv_ (products) − ∆G^ε^_solv_ (reagents) and ∆G^1^ = ∆H^1^ − T∆S^1^ (H and S—enthalpy and entropy, respectively).

A positive ∆G^x^ indicates a lower affinity of the competing metal cation for the HDAC8 complex, whereas a negative value indicates that the formation of the product is thermodynamically favorable, and that the rival metal ion has a higher affinity towards the HDAC8 enzyme site than the one considered a native metal ion (Zn^2+^).

## 4. Conclusions

In this study, we investigate the metal selectivity of a series of divalent biogenic metal ions (M^2+^ = Fe^2+^, Mg^2+^, Co^2+^, and Ni^2+^) for the HDAC8 enzyme site, using Zn^2+^ as a reference. Through a series of modeled metal substitution reactions at the B3LYP/6-31+G(d) level of theory with PCM calculations in methanol and water, we established a hierarchy of metal preferences in the HDAC8 active site. Our results demonstrate that Zn^2+^ remains thermodynamically favorable in all modeled metal–ligand environments and solvents. When increasing their coordination number, metals such as Fe^2+^ and Co^2+^ show some electronic stability, but not enough to overcome entropic and solvation penalties in polar media. Only a limited number of substitution reactions for Fe^2+^ and Co^2+^ yield ∆G values near thermodynamic neutrality, especially for five-coordinate species. However, Zn^2+^ sustains an advantage in even slightly favorable routes, supporting its natural state in the HDAC8 enzyme. Here, computational results match chemical logic derived from HSAB theory and coordination chemistry trends. Zn^2+^, a borderline Lewis acid with a filled d-shell and no ligand-field stabilization, possesses the coordination capability for optimal binding in the HDAC8 environment. Overall, the derived metal substitution hierarchy can be summarized as follows: Zn^2+^ > Co^2+^ ≈ Fe^2+^ > Ni^2+^ > Mg^2+^. These thermodynamic rankings support experimental observations that Zn^2+^ is the biologically relevant cofactor for HDAC8, while Co^2+^ and Fe^2+^ may support limited catalytic activity under specific conditions. However, Mg^2+^ and Ni^2+^ are unlikely to serve as cofactors for HDAC8. These insights are not only important for understanding the biochemical behavior of HDAC8 as a metalloenzyme but also serve as a foundation for guiding the rational design of isoform-specific HDACi. Identifying the most likely native ions is crucial due to the binding mechanism of HDACi—directly to the metal ion in the HDAC8 active site. A deeper understanding of metal selectivity and affinity in the enzyme’s active site can help develop novel, isoform-selective HDACi with an improved therapeutic index.

## Figures and Tables

**Table 1:** Classes and chemical structures of HDACi.

Compound Class	Chemical Structure	Trade Name
Hydroxamic acids	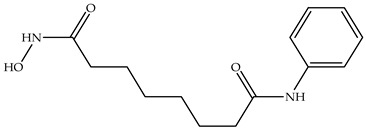	SAHA, Vorinostat
	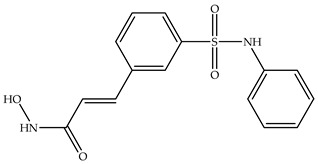	Belinostat
Benzamides	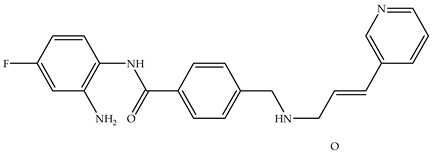	Chidamide
Cyclic tetrapeptides	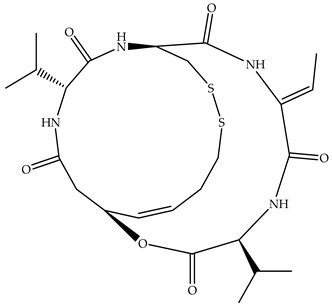	Romidepsin
Short-chain fatty acids	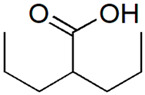	Valproic acid

**Table 2:** Energetics of Fe^2+^–Zn^2+^ substitution in HDAC8 active site models in water and methanol at the B3LYP/6-31+G(d) level of theory. All values are reported in kcal/mol.

Reaction Modeled	ΔE^1^	ΔG^1^	ΔG^32^	ΔG^78^
[Fe(H_2_O)_6_]^2+^ + [Zn-L_4_] + H_2_O → [Zn(H_2_O)_6_]^2+^ + [Fe-L_5_]	−5.67	3.95	10.97	14.59
[Fe(H_2_O)_6_]^2+^ + [Zn-L_4_] + 2H_2_O → [Zn(H_2_O)_6_]^2+^ + [Fe-L_6_]	−23.33	−1.77	15.22	16.62
[Fe(H_2_O)_6_]^2+^ + [Zn-L_5_] → [Zn(H_2_O)_6_]^2+^ + [Fe-L_5_]	4.96	5.11	2.66	6.39
[Fe(H_2_O)_6_]^2+^ + [Zn-L_5_] + H_2_O → [Zn(H_2_O)_6_]^2+^ + [Fe-L_6_]	−12.70	−0.62	6.91	8.42
[Fe(H_2_O)_6_]^2+^ + [Zn-L_6_] → [Zn(H_2_O)_6_]^2+^ + [Fe-L_6_]	11.95	8.40	6.13	8.20
[Fe(H_2_O)_6_]^2+^ + [Zn-L_6_] → [Zn(H_2_O)_6_]^2+^ + [Fe-L_5_] + H_2_O	29.60	14.12	1.88	6.17

**Table 3:** Energetics of Co^2+^–Zn^2+^ substitution in HDAC8 active site models in water and methanol at the B3LYP/6-31+G(d) level of theory. All values are reported in kcal/mol.

Reaction Modeled	ΔE^1^	ΔG^1^	ΔG^32^	ΔG^78^
[Co(H_2_O)_6_]^2+^ + [Zn-L_4_] + H_2_O → [Zn(H_2_O)_6_]^2+^ + [Co-L_5_]	−9.74	−0.96	6.51	8.08
[Co(H_2_O)_6_]^2+^ + [Zn-L_4_] + 2H_2_O → [Zn(H_2_O)_6_]^2+^ + [Co-L_6_]	−18.87	2.32	14.97	15.69
[Co(H_2_O)_6_]^2+^ + [Zn-L_5_] → [Zn(H_2_O)_6_]^2+^ + [Co-L_5_]	0.89	0.19	−1.79	−0.11
[Co(H_2_O)_6_]^2+^ + [Zn-L_5_] + H_2_O → [Zn(H_2_O)_6_]^2+^ + [Co-L_6_]	−8.23	3.47	6.66	7.49
[Co(H_2_O)_6_]^2+^ + [Zn-L_6_] → [Zn(H_2_O)_6_]^2+^ + [Co-L_6_]	16.41	12.48	5.88	7.27
[Co(H_2_O)_6_]^2+^ + [Zn-L_6_] → [Zn(H_2_O)_6_]^2+^ + [Co-L_5_] + H_2_O	25.54	9.20	−2.57	−0.34

**Table 4:** Energetics of Mg^2+^–Zn^2+^ substitution in HDAC8 active site models in water and methanol at B3LYP/6-31+G(d) level of theory. All values are reported in kcal/mol.

Reaction Modeled	ΔE^1^	ΔG^1^	ΔG^32^	ΔG^78^
[Mg(H_2_O)_6_]^2+^ + [Zn-L_4_] + H_2_O → [Zn(H_2_O)_6_]^2+^ + [Mg-L_5_]	11.70	19.48	23.94	26.46
[Mg(H_2_O)_6_]^2+^ + [Zn-L_4_] + 2H_2_O → [Zn(H_2_O)_6_]^2+^ + [Mg-L_6_]	29.75	52.03	65.97	67.65
[Mg(H_2_O)_6_]^2+^ + [Zn-L_5_] → [Zn(H_2_O)_6_]^2+^ + [Mg-L_5_]	22.33	20.63	15.64	18.26
[Mg(H_2_O)_6_]^2+^ + [Zn-L_5_] + H_2_O → [Zn(H_2_O)_6_]^2+^ + [Mg-L_6_]	40.39	53.18	57.66	59.45
[Mg(H_2_O)_6_]^2+^ + [Zn-L_6_] → [Zn(H_2_O)_6_]^2+^ + [Mg-L_6_]	65.03	62.19	56.88	59.23
[Mg(H_2_O)_6_]^2+^ + [Zn-L_6_] → [Zn(H_2_O)_6_]^2+^ + [Mg-L_5_] + H_2_O	46.98	29.64	14.85	18.04

**Table 5:** Energetics of Ni^2+^–Zn^2+^ substitution in HDAC8 active site models in water and methanol at the B3LYP/6-31+G(d) level of theory. All values are reported in kcal/mol.

Reaction Modeled	ΔE^1^	ΔG^1^	ΔG^32^	ΔG^78^
[Ni(H_2_O)_6_]^2+^ + [Zn-L_4_] + H_2_O → [Zn(H_2_O)_6_]^2+^ + [Ni-L_5_]	−5.67	3.95	10.97	14.59
[Ni(H_2_O)_6_]^2+^ + [Zn-L_4_] + 2H_2_O → [Zn(H_2_O)_6_]^2+^ + [Ni-L_6_]	−23.33	−1.77	15.22	16.62
[Ni(H_2_O)_6_]^2+^ + [Zn-L_5_] → [Zn(H_2_O)_6_]^2+^ + [Ni-L_5_]	4.96	5.11	2.66	6.39
[Ni(H_2_O)_6_]^2+^ + [Zn-L_5_] + H_2_O → [Zn(H_2_O)_6_]^2+^ + [Ni-L_6_]	−12.70	−0.62	6.91	8.42
[Ni(H_2_O)_6_]^2+^ + [Zn-L_6_] → [Zn(H_2_O)_6_]^2+^ + [Ni-L_6_]	11.95	8.40	6.13	8.20
[Ni(H_2_O)_6_]^2+^ + [Zn-L_6_] → [Zn(H_2_O)_6_]^2+^ + [Ni-L_5_]+ H_2_O	29.60	14.12	1.88	6.17

## Data Availability

The data presented in this study are available on request from the corresponding authors.

## Supplementary Materials

The following supporting information can be downloaded at: https://www.mdpi.com/article/10.3390/molecules31020306/s1. The Supplementary Materials are available online and include the Cartesian coordinates of all optimized metal–ligand complexes in different dielectric media. These coordinates correspond to the final computed geometries and were used for all structural analysis in the study.

## Abbreviations

The following abbreviations are used in this manuscript:

HDAC	Histone Deacetylase
HDACs	Histone Deacetylases
HATs	Histone Acetyl Transferases
HDAC8	Histone Deacetylase 8
HDACi	Histone Deacetylase Inhibitor
FDA	Food and Drug Administration
PTM	Post-Translational Modification
DFT	Density Functional Theory
PCM	Polarizable Continuum Model
HSAB	Hard and Soft Acids and Bases
B3LYP	Becke, 3-parameter, Lee-Yang-Parr hybrid functional
ZPE	Zero-Point Energy
ΔE^1^	Change in Electronic Energy (gas phase)
ΔG^1^	Change in Gibbs Free Energy (gas phase)
ΔG^32^	Change in Gibbs Free Energy in Methanol (ε = 32)
ΔG^78^	Change in Gibbs Free Energy in Water (ε = 78)
ML_4_	Tetracoordinated Metal–Ligand Complex
ML_5_	Pentacoordinated Metal–Ligand Complex
ML_6_	Hexacoordinated Metal–Ligand Complex
