# Supplementary material for: Metal Preference Hierarchy in the HDAC8 Active Site: A DFT Study
Source: Molecules. 2026 Jan 15;31(2):306. doi: 10.3390/molecules31020306 (PMC12843752; doi:10.3390/molecules31020306)
Supplement: Supplementary file 1 [file molecules-31-00306-s001.zip › molecules-4059768-supplementary.pdf]

Supplementary Information

Table of CONTENTS

1. Computational Details of Modeled Metal-Ligand Complexes -Pages 2-22

Cartesian coordinates of optimized geometries.
$[Fe(H_2O)_6]^{2+}$ in gas phase

O	-1.85499100	0.11260000	1.10149400
H	-1.94712900	0.12649500	2.07120100
O	1.85533800	-0.11196300	-1.10135400
H	2.75431500	-0.15805500	-0.72912700
O	-0.11860400	-2.16976900	0.02098500
H	0.42116100	-2.78663200	-0.50518100
O	1.13484100	-0.04323200	1.80216900
H	1.50406500	0.72712800	2.27112800
O	0.11811000	2.16957300	-0.02109600
H	0.72137100	2.71380700	-0.55830500
O	-1.13494100	0.04324300	-1.80212000
H	-1.50458400	-0.72685400	-2.27117500
Fe	0.00010000	-0.00016000	-0.00004800
H	1.94782500	-0.12600300	-2.07102700
H	-1.41859000	0.83897500	-2.28797400
H	-0.72142200	-2.71376400	0.55893600
H	-2.75413000	0.15803800	0.72958100
H	-0.42251000	2.78617300	0.50449400
H	1.41900800	-0.83875000	2.28807800

Cartesian coordinates of optimized geometries.
$[Fe(H_2O)_6]^{2+}$ in methanol

O	-1.80012000	0.16351600	1.17424600
H	-2.30771000	-0.64820300	1.34803900
O	1.79812300	-0.16541800	-1.17943500
H	1.83201500	-0.68689800	-2.00031700
O	-1.22776900	0.24583100	-1.71952400
H	-0.92907200	0.69252500	-2.53110400
O	-0.31476900	-2.11554300	-0.10549900
H	0.40525300	-2.74439200	0.07756000
O	1.22402500	-0.23725100	1.72350500
H	2.16370800	-0.46655300	1.61615500
O	0.31899100	2.11177900	0.10750500
H	-0.39854200	2.74133300	-0.08264600
Fe	0.00091600	-0.00158700	-0.00051800
H	2.30641500	0.64633100	-1.35087200
H	0.77601600	2.44727300	0.89916300
H	-2.16610300	0.47797000	-1.60526300
H	-1.83560900	0.68834100	1.99288100
H	0.92097300	-0.67833700	2.53645400
H	-0.77900800	-2.45143500	-0.89297500

Cartesian coordinates of optimized geometries.
$[Fe(H_2O)_6]^{2+}$ in water

O	1.79807700	-0.19157800	1.17624800
H	2.30367400	0.61773500	1.36676300
O	-1.79695800	0.19267500	-1.17982600
H	-1.82389700	0.72626300	-1.99322500
O	1.23399000	-0.20350000	-1.72108500
H	0.93736500	-0.63068600	-2.54383800
O	0.31664400	2.11575800	-0.05719400
H	-0.39879800	2.74047400	0.15563000
O	-1.23146100	0.19808500	1.72345800
H	-2.17005400	0.43171000	1.61633000
O	-0.31925500	-2.11332800	0.05925400
H	0.39430100	-2.73848700	-0.15846600
Fe	-0.00066600	0.00108400	-0.00047300
H	-2.30354100	-0.61656100	-1.36785400
H	-0.76288100	-2.46749700	0.85047000
H	2.17164000	-0.43945200	-1.60946000
H	1.82656500	-0.72861000	1.98727400
H	-0.93133700	0.62136900	2.54689900
H	0.76596900	2.47065600	-0.84506700

Cartesian coordinates of optimized geometries.
$[Co(H_2O)_6]^{2+}$ in gas phase

O	-0.00016800	-2.10930700	-0.00594400
H	-0.61580100	-2.68724700	0.47989500
O	0.00019700	2.10930100	0.00598500
H	-0.61471500	2.68485800	0.49554900
O	1.71549100	-0.00412600	1.28357400
H	2.07505100	0.77645300	1.74193200
O	-1.41367600	-0.00477900	1.56317500
H	-2.37770300	-0.00395400	1.42367700
O	-1.71550200	0.00412800	-1.28358300
H	-2.07462100	0.78779900	-1.73698900
O	1.41367600	0.00479400	-1.56317800
H	2.37769900	0.00396500	-1.42365100
H	0.61579600	2.68724000	-0.47989800
H	1.25596300	0.00703400	-2.52418100
H	2.07462000	-0.78779100	1.73698000
H	0.61472500	-2.68486200	-0.49553200
H	-2.07507700	-0.77644400	-1.74194100
H	-1.25593500	-0.00704900	2.52417300
Co	-0.00000500	-0.00000400	-0.00000900

Cartesian coordinates of optimized geometries.
$[Co(H_2O)_6]^{2+}$ in water

O	0.26645600	2.06157400	0.35649800
H	-0.49710300	2.65394400	0.24236500
O	-0.26652800	-2.06170000	-0.35630400
H	-0.75937700	-2.38824000	-1.12903600
O	1.63825200	0.12232300	-1.33750100
H	1.62888000	-0.38465000	-2.16829700
O	-1.47444000	0.54052900	-1.40239300
H	-2.39450800	0.45930400	-1.09513800
O	-1.63825700	-0.12239600	1.33743300
H	-1.90300800	-1.02867200	1.57499200
O	1.47448100	-0.54031100	1.40234900
H	2.39456700	-0.45916700	1.09512800
H	0.49728400	-2.65383200	-0.24260600
H	1.45973200	-0.23936600	2.32714800
H	1.90312900	1.02862000	-1.57484300
H	0.75930900	2.38826300	1.12916100
H	-1.62896500	0.38478700	2.16810200
H	-1.45969700	0.23956400	-2.32718500
Co	0.00000100	-0.00002600	-0.00001700

Cartesian coordinates of optimized geometries.
$[Co(H_2O)_6]^{2+}$ in methanol

O	-0.35441300	2.04554600	-0.36099900
H	0.38061400	2.68110800	-0.30944200
O	0.35443500	-2.04568000	0.36053700
H	0.93378800	-2.34518000	1.08226200
O	-1.64931900	0.05637800	1.33204500
H	-1.63161200	-0.46359500	2.15447900
O	1.44544700	0.60676600	1.40715500
H	2.37038600	0.57088800	1.10619400
O	1.64932400	-0.05626400	-1.33191500
H	1.94460400	-0.94978400	-1.58147900
O	-1.44574700	-0.60632300	-1.40708600
H	-2.37067800	-0.56959400	-1.10621000
H	-0.38052300	-2.68130300	0.30867700
H	-1.43868800	-0.30367500	-2.33148100
H	-1.94374700	0.94995500	1.58240700
H	-0.93366900	2.34474000	-1.08292800
H	1.63133700	0.46303000	-2.15476600
H	1.43868300	0.30372300	2.33142500
Co	0.00006200	-0.00013700	0.00011000

Cartesian coordinates of optimized geometries.
$[Mg(H_2O)_6]^{2+}$ in gas phase

Mg	-0.00007900	-0.00011500	-0.00005900
O	1.62515500	-0.69345800	-1.15297900
H	2.27632300	-1.36904900	-0.89288900
O	-1.62518700	0.69355700	1.15304700
H	-2.27645700	1.36904400	0.89294300
O	-1.22879300	-1.50253200	-0.82677200
H	-2.16860900	-1.66002400	-0.62665900
O	-0.54964300	1.30816500	-1.56079300
H	-0.24642900	2.22508100	-1.68497400
O	1.22874400	1.50243300	0.82669300
H	0.96657000	2.17380600	1.48139300
O	0.54978300	-1.30809000	1.56083100
H	0.24692500	-2.22502300	1.68576100
H	-1.86933400	0.40092100	2.04907600
H	-1.15647100	1.11275700	-2.29678500
H	2.16857200	1.65984700	0.62657400
H	1.86931500	-0.40083700	-2.04900900
H	1.15664700	-1.11190000	2.29658700
H	-0.96657600	-2.17383700	-1.48152500

Cartesian coordinates of optimized geometries.
$[Mg(H_2O)_6]^{2+}$ in water

Mg	-0.00000500	-0.00001300	-0.00004700
O	1.22459500	-1.50781100	-0.74926800
H	1.14086800	-2.45812900	-0.56848600
O	-1.22423800	1.50781000	0.74972800
H	-1.14207900	2.45791500	0.56712200
O	-1.27254000	-0.21735200	-1.63337500
H	-2.07771900	0.29756500	-1.80479500
O	1.10792000	1.41712600	-1.04797000
H	1.88522200	1.89582900	-0.71714800

O	1.27246400	0.21730700	1.63328400
H	1.16052200	0.83966600	2.37014300
O	-1.10799100	-1.41695100	1.04809100
H	-1.88484100	-1.89635000	0.71721500
H	-1.97351600	1.38988200	1.35594600
H	0.93338900	1.73037400	-1.95033000
H	2.07776600	-0.29745900	1.80457500
H	1.97225300	-1.39025500	-1.35755500
H	-0.93297700	-1.73036000	1.95030200
H	-1.16051100	-0.83955900	-2.37035100

Cartesian coordinates of optimized geometries.
$[Mg(H_2O)_6]^{2+}$ in methanol

Mg	-0.00008800	-0.00009400	0.00040100
O	-0.70221400	-1.75971000	-0.86187900
H	-0.17002500	-2.53966800	-1.08830300
O	0.70240000	1.75979600	0.86155800
H	0.17039700	2.53998300	1.08767100
O	-0.34824400	-0.78837700	1.89459000
H	-0.17610900	-0.34519400	2.74140900
O	-1.92908700	0.78406200	-0.02826800
H	-2.32966600	1.29749500	-0.74852800
O	0.34819300	0.78805000	-1.89409600
H	0.71066200	1.66698300	-2.09160200
O	1.92936700	-0.78335700	0.02744100
H	2.33044500	-1.29946700	0.74548800
H	1.62152700	1.95076000	1.10941600
H	-2.59425200	0.69644400	0.67375600
H	0.17367200	0.34579100	-2.74090800
H	-1.62225700	-1.95308700	-1.10441200
H	2.59328300	-0.69499900	-0.67567700
H	-0.70994600	-1.66762700	2.09211800

Cartesian coordinates of optimized geometries.
$[Ni(H_2O)_6]^{2+}$ in gas phase

O	1.41440900	-0.84289200	1.26877200
H	1.31651900	-0.98366800	2.22731800
O	-1.41458000	0.84264700	-1.26879100
H	-2.29528100	1.16599000	-1.00794200
O	0.80057900	1.88487500	0.35919400
H	0.48488100	2.72386100	-0.02114900
O	-1.29790400	0.24439400	1.60463200
H	-1.95561400	-0.40387800	1.91346700
O	-0.80046200	-1.88482300	-0.35922200
H	-1.55612400	-2.08600100	-0.93949800
O	1.29794800	-0.24440400	-1.60458000
H	1.95565200	0.40378800	-1.91359600
H	-1.31663200	0.98380700	-2.22727500
H	1.34992100	-1.02318300	-2.18682800
H	1.55618300	2.08595900	0.93957900
H	2.29524100	-1.16598900	1.00806100
H	-0.48466000	-2.72374200	0.02118000
H	-1.35011200	1.02315000	2.18689000
Ni	0.00000400	0.00005500	-0.00000900

Cartesian coordinates of optimized geometries.
$[Ni(H_2O)_6]^{2+}$ in water

O	-0.35112300	-1.73377400	-1.08399000
H	-0.13604200	-1.76962200	-2.03262200
O	0.35120500	1.73376900	1.08400100
H	1.25641300	2.08115400	0.99206800
O	-1.33865100	1.15870200	-1.08346500
H	-1.18513300	2.11763800	-1.01005600
O	1.66501200	0.56558900	-1.10245000
H	2.41711100	-0.04857300	-1.02771700
O	1.33864000	-1.15873700	1.08347000
H	1.49483900	-0.97500700	2.02649100
O	-1.66506500	-0.56554800	1.10242100
H	-2.41712100	0.04864400	1.02748600
H	0.13612100	1.76967900	2.03262900
H	-1.57738900	-0.78208300	2.04722500
H	-1.49514400	0.97468300	-2.02638100
H	-1.25630100	-2.08123900	-0.99207000
H	1.18520700	-2.11766100	1.00971700
H	1.57723300	0.78232600	-2.04719700
Ni	0.00000200	0.00000200	0.00001900

Cartesian coordinates of optimized geometries.
$[Ni(H_2O)_6]^{2+}$ in methanol

O	-1.07568300	-1.40436300	1.08502900
H	-1.25530700	-1.26036200	2.03076700
O	1.07548100	1.40446000	-1.08505000
H	0.74225500	2.31580700	-1.00147100
O	1.74582700	-0.22962300	1.09846600
H	2.36489300	0.51895700	1.02844300
O	-0.68484300	1.62159000	1.09598700
H	-1.64215400	1.78287000	1.01789600
O	-1.74580300	0.22952500	-1.09854900
H	-1.70535400	0.46887100	-2.04103600
O	0.68501200	-1.62157900	-1.09590600
H	1.64231200	-1.78276200	-1.01746100
H	1.25511900	1.26053700	-2.03079500
H	0.46552500	-1.70528600	-2.04036600
H	1.70543100	-0.46956800	2.04080000
H	-0.74253000	-2.31574000	1.00153400
H	-2.36493000	-0.51896700	-1.02809800
H	-0.46498700	1.70538300	2.04035700
Ni	-0.00000700	0.00000600	-0.00001400

Cartesian coordinates of optimized geometries.
$[Zn(H_2O)_6]^{2+}$ in gas phase

O	0.81821300	1.60351300	1.12756900
H	0.63300800	2.55125200	1.00058400
O	-0.81822500	-1.60351300	-1.12756600
H	-0.63302100	-2.55125400	-1.00059300
O	-1.62800900	-0.12852500	1.35870700
H	-2.36931400	-0.75849200	1.31143100
O	1.09578600	-1.38827700	1.17661100
H	1.99488600	-1.71510400	0.99376200
O	1.62801600	0.12854300	-1.35869800
H	1.76776700	-0.42679600	-2.14653000
O	-1.09577900	1.38827300	-1.17662000
H	-1.99488100	1.71510000	-0.99378000
H	-1.44296300	-1.52598400	-1.87073600
H	0.79364000	-1.81241000	1.99967900
H	2.36931400	0.75851600	-1.31138800
H	1.44295200	1.52599600	1.87074000
H	-0.79362500	1.81240200	-1.99968700
H	-1.76773300	0.42683200	2.14653200
Zn	-0.00000200	-0.00000600	-0.00000100

Cartesian coordinates of optimized geometries.
$[Zn(H_2O)_6]^{2+}$ in water

O	1.27191900	-1.50893400	0.77414900
H	0.90502600	-2.37550300	1.02210700
O	-1.27199100	1.50886100	-0.77390300
H	-0.90525700	2.37562300	-1.02139800
O	1.36341600	0.08648400	-1.62201900
H	1.05391400	0.29573900	-2.52054900
O	1.30377900	1.37754200	0.94965100
H	0.95859600	2.03966200	1.57355300
O	-1.36346500	-0.08630600	1.62195100
H	-1.94985800	0.68630700	1.70456600
O	-1.30366400	-1.37757900	-0.94984500
H	-0.95848800	-2.03989500	-1.57353400
H	-1.84293900	1.23022300	-1.51157200
H	1.90956500	1.84978100	0.35158700
H	-1.05417100	-0.29569400	2.52051900
H	1.84282500	-1.23001400	1.51174800
H	-1.90975600	-1.84953800	-0.35185900
H	1.94996200	-0.68600800	-1.70471300
Zn	0.00002100	-0.00004100	-0.00001100

Cartesian coordinates of optimized geometries.
$[Zn(H_2O)_6]^{2+}$ in methanol

O	1.21950700	-1.66069300	0.48977400
H	0.83076500	-2.55039000	0.55669500
O	-1.21950700	1.66069300	-0.48977600
H	-0.83076800	2.55039100	-0.55669400
O	1.37552200	0.34910900	-1.57440600
H	1.08667100	0.74788200	-2.41385000
O	1.32877300	1.14751300	1.18984100
H	1.00334800	1.70055000	1.92122500
O	-1.37552400	-0.34910600	1.57440500
H	-1.94441700	0.40616900	1.80635900
O	-1.32877100	-1.14751500	-1.18983900
H	-1.00334400	-1.70055600	-1.92121900
H	-1.79036300	1.54912900	-1.27067800
H	1.96911500	1.68857200	0.69515900
H	-1.08667500	-0.74787600	2.41385100
H	1.79036600	-1.54912900	1.27067400
H	-1.96911600	-1.68857000	-0.69515600
H	1.94441400	-0.40616500	-1.80636400
Zn	0.00000000	0.00000000	0.00000100

Cartesian coordinates of optimized geometries.
H_2O in gas phase

O	0.00000000	0.00000000	0.11729000
H	0.00000000	0.77128800	-0.46915900
H	0.00000000	-0.77128800	-0.46915900

Cartesian coordinates of optimized geometries.
H_2O in water

O	0.00000000	0.00000000	0.11857400
H	0.00000000	0.76844900	-0.47429600
H	0.00000000	-0.76844900	-0.47429600

Cartesian coordinates of optimized geometries.
H_2O in methanol

O	0.00000000	0.00000000	0.11857400
H	0.00000000	0.76844900	-0.47429600
H	0.00000000	-0.76844900	-0.47429600

Cartesian coordinates of optimized geometries.
[Zn-L$_4$]: Metal-ligand environment involving Asp178, Asp267, His180, and the Lys backbone in gas phase

Zn	0	-0.63607200	-0.72127400	-0.56623800
O	0	-1.68009600	0.15922200	-1.91277800
O	0	-1.43829600	-1.87830000	0.73112200
C	0	0.56061200	1.60389700	1.13324300
O	0	-0.36138900	0.82673600	0.85542200
C	-1	0.73079000	2.20766400	2.48527300
H	0	-0.11236100	1.89799900	3.10869000
H	0	1.64536300	1.87091000	2.98152200
H	0	0.74133600	3.30021800	2.44953600
N	0	1.52743900	1.98401600	0.29031100
H	0	1.45869000	1.76293500	-0.69405700
C	0	-2.60620000	-1.49888500	1.25597700
O	0	-1.77896500	2.33560200	-1.34635300
O	0	-3.06664200	-2.00114700	2.27354700
C	0	-2.31762700	1.23891500	-1.49029600
C	-1	-3.31793700	-0.37585300	0.44035900
C	-1	-3.81796000	1.09739100	-1.17646300
H	0	-4.32704900	0.19359900	-1.47582400
H	0	-4.12168500	1.50315100	-0.22352100
H	0	-4.24201300	1.85568000	-1.87787800
H	0	-4.34896500	-0.29885400	0.78090600
H	0	-2.74706200	0.51257500	0.67441800
H	0	-3.22129200	-0.74709400	-0.55805900
N	0	1.38755200	-1.24338700	-0.68320800
C	0	1.86915200	-1.87299300	0.37781700
C	0	2.47561800	-0.67059900	-1.32590800
H	0	1.27167200	-2.41320500	1.10120500
C	0	3.63252200	-0.93798200	-0.63381000
H	0	2.35379000	-0.11020700	-2.24287900
H	0	3.81392700	-2.11623900	1.15134200
N	0	3.21448900	-1.71289800	0.44265200
C	-1	5.05765200	-0.49954400	-0.82150800
H	0	5.74146100	-1.35322800	-0.90539600
H	0	5.14602000	0.08714800	-1.74002600
H	0	5.39843300	0.12572200	0.01310500
C	-1	2.64104400	2.71989100	0.82922200
H	0	3.35153500	2.90762800	0.01724200
H	0	2.35460900	3.69745700	1.22639000
H	0	3.17652200	2.15957600	1.60102900

Cartesian coordinates of optimized geometries.
[Zn-L$_4$]: Metal-ligand environment involving Asp178, Asp267, His180, and the Lys backbone in water

Zn	0	-0.54967400	-0.70637700	-0.59675700
O	0	-1.77965600	0.20075500	-1.85818900
O	0	-1.49547200	-1.90816600	0.67484600
C	0	0.57672000	1.52765400	1.22234200
O	0	-0.36869600	0.76956100	0.90119500
C	-1	0.74845300	2.06962600	2.60132400
H	0	-0.09704500	1.74787700	3.21564000
H	0	1.66083400	1.69291900	3.07135900
H	0	0.78116700	3.16116900	2.61764700
N	0	1.53871400	1.93253700	0.40153200
H	0	1.56599200	1.62829500	-0.56310200
C	0	-2.65325300	-1.55790700	1.17774600
O	0	-1.85474000	2.41857000	-1.50383500
O	0	-3.16769000	-2.14948400	2.14382700
C	0	-2.38734600	1.29910500	-1.48993500
C	-1	-3.33176700	-0.37403100	0.44983300
C	-1	-3.86039200	1.20700100	-1.05198400
H	0	-4.43002500	0.33327500	-1.32839900
H	0	-4.06449400	1.58812700	-0.06188500
H	0	-4.31423500	2.00212500	-1.68754000
H	0	-4.35474200	-0.27342100	0.80592300
H	0	-2.71812600	0.47589700	0.71627000
H	0	-3.25064800	-0.68814500	-0.57013400
N	0	1.40840500	-1.27088100	-0.64454900
C	0	1.96543500	-1.85883300	0.40917100
C	0	2.44329100	-0.67101700	-1.35118900
H	0	1.44288700	-2.41169900	1.17566000
C	0	3.63420300	-0.88433700	-0.70185000
H	0	2.26223900	-0.12677900	-2.26729400
H	0	3.94480800	-2.00321900	1.09859600
N	0	3.29808300	-1.65040500	0.40246600
C	-1	5.02141100	-0.39193600	-0.95788700
H	0	5.73765300	-1.21887600	-1.02947300
H	0	5.04977800	0.16640500	-1.89715000
H	0	5.35588700	0.27405800	-0.15385800
C	-1	2.62997800	2.69980300	0.95307300
H	0	3.33990900	2.90157000	0.14639400
H	0	2.30170500	3.66658000	1.34000800
H	0	3.16988400	2.15574400	1.73127100

Cartesian coordinates of optimized geometries.
[Zn-L_4]: Metal-ligand environment involving Asp178, Asp267, His180, and the Lys backbone in methanol

Zn	0	-0.55381800	-0.70617500	-0.59586300
O	0	-1.77600600	0.20309400	-1.85978000
O	0	-1.49375500	-1.90876100	0.67408100
C	0	0.57684900	1.52736600	1.22232100
O	0	-0.36975500	0.77222300	0.89969000
C	-1	0.75046700	2.06563900	2.60243500
H	0	-0.09549600	1.74412000	3.21616000
H	0	1.66218200	1.68590400	3.07137800
H	0	0.78535300	3.15708200	2.62132400
N	0	1.53988500	1.93179300	0.40210900
H	0	1.56341000	1.63275900	-0.56420300
C	0	-2.65207100	-1.55831400	1.17766800
O	0	-1.85183800	2.41996800	-1.49974300
O	0	-3.16551900	-2.14889600	2.14401600
C	0	-2.38449000	1.30096500	-1.48904300
C	-1	-3.33063800	-0.37470000	0.44871100
C	-1	-3.85799200	1.20734000	-1.05245100
H	0	-4.42680900	0.33353500	-1.33020300
H	0	-4.06310800	1.58786500	-0.06236200
H	0	-4.31143800	2.00273400	-1.68795600
H	0	-4.35389300	-0.27469100	0.80418100
H	0	-2.71774800	0.47550700	0.71588400
H	0	-3.24904300	-0.68872200	-0.57127400
N	0	1.40649500	-1.26507500	-0.64677100
C	0	1.96123600	-1.85701000	0.40577500
C	0	2.44348100	-0.66657100	-1.35148900
H	0	1.43628400	-2.41035400	1.17032100
C	0	3.63358900	-0.88456200	-0.70215000
H	0	2.26457300	-0.12079400	-2.26713100
H	0	3.93994500	-2.00939700	1.09554100
N	0	3.29457600	-1.65199700	0.40054700
C	-1	5.02295300	-0.39672700	-0.95688000
H	0	5.73560800	-1.22633100	-1.03316500
H	0	5.05314600	0.16570900	-1.89365000
H	0	5.36118900	0.26409900	-0.15014900
C	-1	2.63277500	2.69547300	0.95495300
H	0	3.34293700	2.89806200	0.14860200
H	0	2.30688700	3.66209600	1.34436200
H	0	3.17190600	2.14861600	1.73180900

Cartesian coordinates of optimized geometries.
[Zn-L_5]: Metal-ligand environment involving one water molecule, Asp178, Asp267, His180, and the Lys backbone in gas phase

O	0	-1.98740400	-0.23654000	-1.75520900
O	0	-1.86749900	-0.60060600	1.55819700
C	0	1.96144600	2.01135500	0.60695300
O	0	0.98771400	1.45616100	1.10625000
C	-1	2.33541200	3.45509100	0.80494500
H	0	1.61081100	3.90439600	1.49026300
H	0	3.32929600	3.57867200	1.24365000
H	0	2.29816000	4.01653400	-0.13243500
N	0	2.86506900	1.38495900	-0.19630700
H	0	2.58330900	0.50975300	-0.62304300
C	0	-2.55969100	0.51282000	1.81216200
O	0	-0.74305400	1.60433000	-2.12858000
O	0	-2.93220700	0.81498400	2.93715700
C	0	-1.81842500	1.07285500	-1.86404100
C	-1	-2.86207000	1.34768000	0.53708200
C	-1	-3.05336500	1.94094700	-1.61864300
H	0	-3.99889200	1.45236600	-1.42964100
H	0	-2.87318600	2.82615400	-1.02749700
H	0	-3.21005200	2.38294900	-2.63348300
H	0	-3.56945300	2.13504600	0.79221400
H	0	-1.89301300	1.74585300	0.26202200
H	0	-3.27312400	0.60304900	-0.11060400
N	0	0.69310900	-1.68220600	0.07437600
C	0	1.22030400	-1.55076900	1.28226900
C	0	1.74133000	-1.93300800	-0.79710600
H	0	0.66708100	-1.29278800	2.17463400
C	0	2.92478200	-1.96000300	-0.10197400
H	0	1.57373600	-2.06916100	-1.85593400
H	0	3.19163200	-1.62867400	2.00143700
N	0	2.55824700	-1.72931600	1.21916100
C	-1	4.34735300	-2.12520300	-0.53554700
H	0	4.81948700	-2.99814900	-0.06724000
H	0	4.39116100	-2.26644900	-1.61915000
H	0	4.94884400	-1.24256300	-0.28443300
C	-1	3.87951000	2.19045800	-0.82925000
H	0	4.49085300	1.53612400	-1.46180300
H	0	3.47671400	2.97264700	-1.48291500
H	0	4.55774400	2.64288900	-0.10291000
O	0	-2.50127800	-2.74082000	-0.70089800
H	0	-3.14300200	-2.83767600	0.02339100
H	0	-2.96354900	-2.26495900	-1.41757900
Zn	0	-1.20597400	-1.05578300	-0.17786600

Cartesian coordinates of optimized geometries.
[Zn-L_5]: Metal-ligand environment involving one water molecule, Asp178, Asp267, His180, and the Lys backbone in water

O	0	-2.05039300	-0.12550800	-1.69971500
O	0	-1.87375300	-0.64466500	1.55528200
C	0	1.80531900	2.15880000	0.56415600
O	0	0.72054100	1.65150900	0.88096100
C	-1	2.34342600	3.43714000	1.13450600
H	0	1.61890200	3.83300900	1.85283100
H	0	3.28807800	3.28928500	1.66432700
H	0	2.50076200	4.20071400	0.36825600
N	0	2.66078100	1.64065600	-0.33614600
H	0	2.45816700	0.74785700	-0.77119300
C	0	-2.53908900	0.44858100	1.83582400
O	0	-0.85871300	1.63610100	-2.42905100
O	0	-2.89822400	0.72840400	2.99333500
C	0	-1.87101000	1.15423300	-1.89991200
C	-1	-2.84024500	1.34291200	0.61362800
C	-1	-3.00075800	2.10984500	-1.48914800
H	0	-3.96856400	1.69640500	-1.25125500
H	0	-2.69338500	2.93365900	-0.86085400
H	0	-3.19424400	2.63329100	-2.45509300
H	0	-3.52247900	2.14048000	0.90133900
H	0	-1.85874200	1.71543900	0.35010900
H	0	-3.26098900	0.63452300	-0.07054200
N	0	0.74161900	-1.71285100	0.05380100
C	0	1.27093100	-1.86035800	1.26148500
C	0	1.79503300	-1.76015600	-0.84940000
H	0	0.72922600	-1.85530900	2.19526900
C	0	2.97728600	-1.93506700	-0.17126700
H	0	1.63336300	-1.66733500	-1.91357000
H	0	3.23894300	-2.12796900	1.94648300
N	0	2.60901600	-2.00004300	1.16311300
C	-1	4.39982000	-2.00888400	-0.62550500
H	0	4.86605300	-2.95698900	-0.33270400
H	0	4.44597200	-1.92899600	-1.71475300
H	0	4.99567200	-1.19466100	-0.19697800
C	-1	3.91800200	2.31493400	-0.57316400
H	0	4.47036500	1.74187200	-1.32411200
H	0	3.78263800	3.32109200	-0.97672700
H	0	4.54716900	2.36329400	0.31999000
O	0	-2.35040900	-2.90885300	-0.60655100
H	0	-2.83303600	-3.19386600	0.18898500
H	0	-3.03426300	-2.71818600	-1.27254700
Zn	0	-1.17847400	-1.14032500	-0.23913100

Cartesian coordinates of optimized geometries.
[Zn-L_5]: Metal-ligand environment involving one water molecule, Asp178, Asp267, His180, and the Lys backbone in methanol

O	0	-2.04900800	-0.12941100	-1.70169600
O	0	-1.87611000	-0.64085500	1.55518200
C	0	1.81101100	2.14986300	0.55640800
O	0	0.72345000	1.64966100	0.87347400
C	-1	2.35415700	3.42894200	1.12061000
H	0	1.63067900	3.83110200	1.83642300
H	0	3.29797100	3.28047000	1.65175700
H	0	2.51445600	4.18790500	0.35038300
N	0	2.66734200	1.62230000	-0.33817400
H	0	2.45583900	0.73424900	-0.77854300
C	0	-2.53898800	0.45524400	1.83469500
O	0	-0.85114600	1.62906700	-2.42916100
O	0	-2.89940400	0.73595500	2.99080200
C	0	-1.86562500	1.15045100	-1.90261100
C	-1	-2.83628600	1.34914000	0.61067000
C	-1	-2.99510900	2.10726600	-1.49544100
H	0	-3.96358500	1.69486300	-1.25831700
H	0	-2.68822100	2.93250000	-0.86888600
H	0	-3.18677800	2.62868600	-2.46294500
H	0	-3.51727900	2.14844900	0.89648900
H	0	-1.85388000	1.71956700	0.34731200
H	0	-3.25804500	0.64040500	-0.07233000
N	0	0.73652500	-1.70469100	0.05771600
C	0	1.26708000	-1.81116900	1.26905200
C	0	1.78825400	-1.78531800	-0.84495400
H	0	0.72603700	-1.76954200	2.20229200
C	0	2.97093600	-1.93927500	-0.16281900
H	0	1.62542600	-1.72821900	-1.91143000
H	0	3.23541300	-2.05896300	1.96006600
N	0	2.60463200	-1.95721700	1.17367200
C	-1	4.39244900	-2.03135800	-0.61600300
H	0	4.85834500	-2.96847300	-0.28911500
H	0	4.43735200	-1.99190300	-1.70752300
H	0	4.98995700	-1.20253300	-0.21872300
C	-1	3.92454200	2.29413900	-0.58245000
H	0	4.47584700	1.71467900	-1.32934500
H	0	3.78972600	3.29723500	-0.99405800
H	0	4.55471400	2.34931900	0.30955100
O	0	-2.35216400	-2.90983000	-0.59774500
H	0	-2.83990900	-3.18548400	0.19796400
H	0	-3.03156300	-2.72487800	-1.26987900
Zn	0	-1.18371600	-1.13788100	-0.23644800

Cartesian coordinates of optimized geometries.
[Zn-L$_6$]: Metal-ligand environment involving two water molecules, Asp178, Asp267, His180, and the Lys backbone in gas phase

Zn	0	-0.73566800	-0.57679600	-0.36043900
O	0	-1.51632100	1.02189700	-1.23413600
O	0	-1.74598000	-2.15802100	-0.00893700
C	0	0.78689600	0.47552500	2.12663100
O	0	-0.27281000	0.13786600	1.54801100
C	-1	1.01000400	0.24985900	3.59079200
H	0	0.11748000	-0.21866200	4.01485600
H	0	1.85581800	-0.41459300	3.78721300
H	0	1.18533500	1.19039700	4.11997000
N	0	1.81927200	1.06878200	1.53231800
H	0	1.67086000	1.61546900	0.66163400
C	0	-3.02818800	-2.00024600	0.30098000
O	0	-1.59723200	2.80817700	0.12137000
O	0	-3.75951600	-2.91789100	0.64717800
C	0	-2.15742500	1.86048000	-0.43816300
C	-1	-3.49054900	-0.52458500	0.15611600
C	-1	-3.66828700	1.67677500	-0.24010000
H	0	-4.22022900	1.13771200	-0.98950900
H	0	-3.97557100	1.50953100	0.78141100
H	0	-4.00812800	2.72679600	-0.39231200
H	0	-4.55677800	-0.47275800	0.36382400
H	0	-2.90429300	-0.00806700	0.90422300
H	0	-3.23501500	-0.30091900	-0.86283600
N	0	1.05985700	-1.22396700	-1.01716300
C	0	1.53743600	-2.36360400	-0.53844300
C	0	2.14440200	-0.44509900	-1.37211100
H	0	0.93790500	-3.18530600	-0.17063100
C	0	3.30188500	-1.11163400	-1.08078700
H	0	2.00893000	0.53228100	-1.81201900
H	0	3.49745500	-3.09063300	-0.27092500
N	0	2.89162000	-2.33443400	-0.56190900
C	-1	4.71931100	-0.66804600	-1.14038000
H	0	5.34880200	-1.34440900	-1.73337400
H	0	4.77340800	0.32167600	-1.60208700
H	0	5.16275700	-0.59364300	-0.13872300
C	-1	2.99664700	1.30882800	2.33151600
H	0	3.74353200	1.78649200	1.68917100
H	0	2.82421300	1.98895900	3.17240800
H	0	3.43677700	0.38071700	2.70509800
O	0	1.16709400	2.95924400	-0.37904800
H	0	1.13547300	2.82422700	-1.34851200
H	0	0.20984300	3.02091700	-0.13529400
O	0	0.46674700	1.96017600	-2.90325900
H	0	0.15756600	2.42240100	-3.69599200
H	0	-0.34001300	1.66151600	-2.41287900

Cartesian coordinates of optimized geometries.
[Zn-L$_6$]: Metal-ligand environment involving two water molecules, Asp178, Asp267, His180, and the Lys backbone in water

Zn	0	-0.66124300	-0.55931900	-0.42207900
O	0	-1.60000300	1.09030000	-1.09296300
O	0	-1.75958800	-2.17599300	-0.18038100
C	0	0.79877400	0.29064700	2.14705400
O	0	-0.27068400	-0.02096200	1.54950500
C	-1	1.05383900	-0.07247600	3.57364900
H	0	0.17370600	-0.58231400	3.97575200
H	0	1.90275800	-0.75370300	3.67673800
H	0	1.24780400	0.80900800	4.18896300
N	0	1.80043400	0.96828900	1.60578100
H	0	1.66505400	1.51010500	0.74120000
C	0	-3.02489400	-2.03838200	0.12337400
O	0	-1.65057700	2.78784400	0.36907900
O	0	-3.77378300	-3.00207800	0.35071800
C	0	-2.21958800	1.86431100	-0.23717600
C	-1	-3.49234500	-0.56939900	0.14773300
C	-1	-3.72184900	1.65757500	-0.00379100
H	0	-4.29644600	1.18968800	-0.78407600
H	0	-3.99283900	1.38046200	1.00509300
H	0	-4.07697700	2.71141000	-0.03885800
H	0	-4.55268200	-0.54203600	0.38480600
H	0	-2.88799700	-0.12989400	0.92908100
H	0	-3.25873200	-0.23617600	-0.84635400
N	0	1.10754000	-1.24153900	-1.04861500
C	0	1.69665900	-2.31351600	-0.52802300
C	0	2.11664400	-0.39287800	-1.46739300
H	0	1.19637200	-3.17036800	-0.10208200
C	0	3.32886000	-0.94940200	-1.17356000
H	0	1.89808300	0.55944700	-1.92750600
H	0	3.71214500	-2.85414600	-0.27003900
N	0	3.03619700	-2.17219600	-0.59399700
C	-1	4.69338300	-0.38989600	-1.28824800
H	0	5.36779900	-1.04840100	-1.84948400
H	0	4.65560200	0.57310500	-1.80436200
H	0	5.13960000	-0.22601100	-0.29952400
C	-1	2.99511100	1.16050600	2.40517200
H	0	3.72629500	1.68918300	1.78722000
H	0	2.81785400	1.77293600	3.29370400
H	0	3.44520300	0.21080700	2.69794200
O	0	1.08256400	3.01375700	-0.27182300
H	0	1.02248800	2.85429500	-1.23823400
H	0	0.13574600	2.98058100	0.01128300
O	0	0.27444400	2.18504100	-2.85131900
H	0	-0.08708700	2.85944000	-3.44792600
H	0	-0.49221300	1.83444500	-2.33840900

Cartesian coordinates of optimized geometries.
[Zn-L_6]: Metal-ligand environment involving two water molecules, Asp178, Asp267, His180, and the Lys backbone in methanol

Zn	0	-0.66489800	-0.56247300	-0.41704400
O	0	-1.59266100	1.08718500	-1.09690300
O	0	-1.76098000	-2.17669600	-0.17278800
C	0	0.79801800	0.29976600	2.14698300
O	0	-0.27164900	-0.01051600	1.54997100
C	-1	1.05206900	-0.06042100	3.57463600
H	0	0.17056200	-0.56708200	3.97768300
H	0	1.89933500	-0.74338100	3.67991000
H	0	1.24783100	0.82231100	4.18760200
N	0	1.80149900	0.97339300	1.60364100
H	0	1.66598000	1.51662000	0.73959800
C	0	-3.02690700	-2.03619700	0.12999100
O	0	-1.64731200	2.79259500	0.35570500
O	0	-3.77682500	-2.99726000	0.36145300
C	0	-2.21487400	1.86578100	-0.24612900
C	-1	-3.49187900	-0.56601000	0.14699000
C	-1	-3.71798800	1.66050300	-0.01467300
H	0	-4.29147400	1.19011300	-0.79424100
H	0	-3.99076900	1.38765700	0.99484400
H	0	-4.07203800	2.71451600	-0.05434600
H	0	-4.55257900	-0.53617900	0.38203500
H	0	-2.88842300	-0.12415000	0.92774700
H	0	-3.25610500	-0.23737200	-0.84811600
N	0	1.10436000	-1.23855500	-1.04581600
C	0	1.68808200	-2.31627800	-0.53116200
C	0	2.11768900	-0.39297900	-1.46063200
H	0	1.18301100	-3.17292300	-0.11042300
C	0	3.32735200	-0.95698200	-1.17000500
H	0	1.90391500	0.56193900	-1.91759100
H	0	3.70099400	-2.86866300	-0.27697100
N	0	3.02843600	-2.18116200	-0.59613500
C	-1	4.69550600	-0.40476700	-1.28255000
H	0	5.36658400	-1.06593500	-1.84460000
H	0	4.66346000	0.55928000	-1.79708300
H	0	5.14192600	-0.24475400	-0.29323400
C	-1	2.99620600	1.16447300	2.40248100
H	0	3.72895100	1.68977100	1.78343200
H	0	2.82092600	1.77968100	3.28954900
H	0	3.44394400	0.21441900	2.69796800
O	0	1.08770800	3.01158100	-0.27766900
H	0	1.02790600	2.85354300	-1.24430700
H	0	0.14029500	2.98482400	0.00419700
O	0	0.28347800	2.17667300	-2.85469600
H	0	-0.07627800	2.84641500	-3.45752200
H	0	-0.48478600	1.82797500	-2.34280700

Cartesian coordinates of optimized geometries.
[Fe-L_5]: Metal-ligand environment involving one water molecule, Asp178, Asp267, His180, and the Lys backbone in gas phase.

O	0	-1.95890000	-0.26029400	-1.77079200
O	0	-1.91425500	-0.70591500	1.57581200
C	0	1.83637000	2.03534300	0.54616900
O	0	0.80602800	1.49629400	0.94001600
C	-1	2.28062400	3.42929700	0.90479400
H	0	1.54254800	3.85714400	1.58952700
H	0	3.25081800	3.44646300	1.40878900
H	0	2.33521300	4.07898200	0.02722700
N	0	2.74919300	1.43655200	-0.26483100
H	0	2.46159000	0.60232500	-0.76378000
C	0	-2.59066000	0.42012100	1.84692100
O	0	-0.78845200	1.61332900	-2.20084400
O	0	-2.94080500	0.72822300	2.97453700
C	0	-1.83266600	1.05324200	-1.87983500
C	-1	-2.88270000	1.24907700	0.56705400
C	-1	-3.07743100	1.89443600	-1.57319900
H	0	-4.01282400	1.38997700	-1.37781900
H	0	-2.89152000	2.76656600	-0.96482100
H	0	-3.26153000	2.36036800	-2.57151800
H	0	-3.60128100	2.02839100	0.81410600
H	0	-1.91184600	1.65421700	0.30661600
H	0	-3.27376700	0.50108500	-0.09087200
N	0	0.73139900	-1.74692300	0.10953900
C	0	1.28171200	-1.60001400	1.30500000
C	0	1.77140900	-1.97152100	-0.77829000
H	0	0.74480900	-1.36499800	2.21331700
C	0	2.96955900	-1.95895200	-0.10817600
H	0	1.58962400	-2.12055100	-1.83336100
H	0	3.27219700	-1.63030200	1.98828200
N	0	2.62625900	-1.73448800	1.21710900
C	-1	4.38305100	-2.08270500	-0.57237300
H	0	4.89723500	-2.93203000	-0.10439800
H	0	4.40679900	-2.23870300	-1.65471000
H	0	4.96207800	-1.17726100	-0.34983300
C	-1	3.84853000	2.23117000	-0.75660600
H	0	4.45634700	1.60172700	-1.41693200
H	0	3.53346500	3.09662000	-1.35050700
H	0	4.50664900	2.56787700	0.04737600
Fe	0	-1.22687100	-1.10677800	-0.15177300
O	0	-2.63123900	-2.69439000	-0.85436300
H	0	-3.31823600	-2.89156900	-0.19663000
H	0	-3.04349000	-2.12103300	-1.53034700

Cartesian coordinates of optimized geometries.
[Fe-L$_5$]: Metal-ligand environment involving one water molecule, Asp178, Asp267, His180, and the Lys backbone in water

O	0	-1.96223500	-0.11564900	-1.76626700
O	0	-1.96129000	-0.75262300	1.53531900
C	0	1.75206200	2.17314900	0.67327900
O	0	0.74181300	1.57267900	1.06741600
C	-1	2.16329600	3.53799300	1.13799200
H	0	1.43000100	3.89661000	1.86692200
H	0	3.14103300	3.53278200	1.62663300
H	0	2.19713600	4.26238100	0.31987700
N	0	2.62741500	1.69212600	-0.22753500
H	0	2.50467500	0.75641900	-0.59879100
C	0	-2.62227900	0.34657000	1.82959200
O	0	-0.90579100	1.77974000	-2.34441500
O	0	-2.96602800	0.62608600	2.98743900
C	0	-1.89519000	1.18327100	-1.89367000
C	-1	-2.93096800	1.23788000	0.60394400
C	-1	-3.12269500	2.01012800	-1.49417400
H	0	-4.05316800	1.49844100	-1.30071800
H	0	-2.91525000	2.84467800	-0.84048500
H	0	-3.33708000	2.53604900	-2.45452000
H	0	-3.65480900	2.00007600	0.88372300
H	0	-1.96131300	1.65886500	0.36474200
H	0	-3.30548400	0.51806100	-0.09431400
N	0	0.76411000	-1.82467300	0.02484300
C	0	1.30166900	-2.14016700	1.19514400
C	0	1.82501100	-1.67151500	-0.85814500
H	0	0.76349400	-2.32128400	2.11384700
C	0	3.01581400	-1.88961100	-0.20549000
H	0	1.66452600	-1.41924200	-1.89698000
H	0	3.28344700	-2.40924600	1.85459100
N	0	2.65016300	-2.18911700	1.09501700
C	-1	4.43942800	-1.80914900	-0.65429400
H	0	4.96498400	-2.75844400	-0.49614200
H	0	4.47997400	-1.57199000	-1.72075500
H	0	4.98528000	-1.02963300	-0.10957600
C	-1	3.78199300	2.49096100	-0.57596100
H	0	4.36536400	1.93394000	-1.31539500
H	0	3.50760200	3.44243900	-1.03754100
H	0	4.43995800	2.67701900	0.27740000
Fe	0	-1.23547400	-1.16986700	-0.25313000
O	0	-2.18986100	-3.01900300	-0.79679800
H	0	-1.81833200	-3.84181000	-0.43588800
H	0	-3.15324500	-3.08482400	-0.68238600

Cartesian coordinates of optimized geometries.
[Fe-L$_5$]: Metal-ligand environment involving one water molecule, Asp178, Asp267, His180, and the Lys backbone in methanol

O	0	-1.95032200	-0.13674700	-1.759100
O	0	-1.95670200	-0.76400800	1.54859300
C	0	1.72141700	2.17687000	0.63858200
O	0	0.70482500	1.58308100	1.02512800
C	-1	2.14255600	3.53718000	1.10987800
H	0	1.40860500	3.89917900	1.83656200
H	0	3.11780400	3.52263800	1.60276800
H	0	2.18526500	4.26380000	0.29381900
N	0	2.59848800	1.69235700	-0.25948100
H	0	2.46596800	0.76385700	-0.64423000
C	0	-2.62661800	0.33210700	1.83893900
O	0	-0.93454400	1.77070500	-2.36823200
O	0	-2.97025300	0.61455200	2.99529400
C	0	-1.90715400	1.16255800	-1.89905100
C	-1	-2.94458300	1.21417900	0.60821200
C	-1	-3.14551200	1.97013500	-1.49492300
H	0	-4.06703800	1.44436900	-1.29851600
H	0	-2.94873700	2.80901800	-0.84341500
H	0	-3.37076800	2.49156100	-2.45520600
H	0	-3.67923300	1.96771300	0.88290100
H	0	-1.98007300	1.64669200	0.36854800
H	0	-3.30808800	0.48544600	-0.08683000
N	0	0.76598100	-1.79282800	0.06917100
C	0	1.31720500	-2.00718800	1.25538600
C	0	1.81637300	-1.70718600	-0.83494200
H	0	0.78904300	-2.11074100	2.19166300
C	0	3.01552400	-1.86476700	-0.17967300
H	0	1.64364100	-1.53817400	-1.88851500
H	0	3.30729800	-2.21090400	1.91360500
N	0	2.66506600	-2.05824800	1.14521500
C	-1	4.43342600	-1.81437600	-0.64977200
H	0	4.96498700	-2.74745100	-0.42614000
H	0	4.46063500	-1.65982500	-1.73172700
H	0	4.98277000	-0.99424900	-0.17312500
C	-1	3.76003400	2.48355900	-0.60118400
H	0	4.34235200	1.92483800	-1.34001900
H	0	3.49547300	3.43905400	-1.06075600
H	0	4.41633400	2.66212400	0.25537100
Fe	0	-1.23698100	-1.17742900	-0.23867700
O	0	-2.03935300	-3.08230900	-0.85907000
H	0	-1.60190600	-3.87915200	-0.51411300
H	0	-2.99476000	-3.23621600	-0.76580300

Cartesian coordinates of optimized geometries.
[Fe-L$_6$]: Metal-ligand environment involvivng two water molecules, Asp178, Asp267, His180, and the Lys backbone in gas phase

O	0	-1.52551300	0.60626100	-2.00453600
O	0	-2.00416000	-0.38359700	1.21501000
C	0	2.30982900	1.66316600	0.91742200
O	0	1.15269500	1.31325700	1.13324100
C	-1	2.99315000	2.83515100	1.56773500
H	0	2.28953700	3.29577100	2.26734700
H	0	3.88291000	2.54617400	2.13395800
H	0	3.28150400	3.59666700	0.83804700
N	0	3.15959200	1.02507300	0.07007800
H	0	2.76499300	0.37244100	-0.59777200
C	0	-2.46553600	0.81503000	1.62361900
O	0	-0.01595300	2.27550600	-2.01861900
O	0	-2.88892300	0.99713500	2.75290800
C	0	-1.16545400	1.87312100	-1.86781000
C	-1	-2.43372900	1.87435300	0.49360400
C	-1	-2.27301400	2.87221900	-1.50977600
H	0	-3.29700600	2.52811700	-1.48599700
H	0	-2.00607800	3.58150100	-0.74104300
H	0	-2.26150900	3.52164900	-2.41771200
H	0	-3.00686500	2.73964900	0.81937900
H	0	-1.37824000	2.09689300	0.38188000
H	0	-2.88899400	1.33748400	-0.31316800
N	0	0.56125200	-1.65085100	-0.21303900
C	0	1.02492000	-1.75217100	1.02161400
C	0	1.60594800	-2.00260300	-1.05345700
H	0	0.47582700	-1.49870800	1.91691100
C	0	2.71973400	-2.31840700	-0.31365700
H	0	1.48883000	-2.00000500	-2.12805600
H	0	2.90083400	-2.27967800	1.82140800
N	0	2.31567100	-2.16569700	1.00468000
C	-1	4.11450400	-2.69693800	-0.69588600
H	0	4.39437400	-3.68390100	-0.30557400
H	0	4.20412000	-2.73532700	-1.78530600
H	0	4.84716900	-1.96956100	-0.32340900
C	-1	4.46009000	1.60722600	-0.16271300
H	0	4.98860600	0.98068200	-0.89039500
H	0	4.41873900	2.61593000	-0.58857600
H	0	5.07337600	1.62408200	0.74117500
Fe	0	-1.24971200	-0.64864800	-0.57590700
O	0	-2.56740200	-2.26133000	-1.22926500
H	0	-3.05863200	-2.54883000	-0.41009100
H	0	-3.23918400	-1.93344400	-1.84857300
O	0	-3.67444100	-2.48934200	1.19329300
H	0	-3.13295000	-1.71384200	1.49892300
H	0	-3.65753000	-3.15256600	1.89776700

Cartesian coordinates of optimized geometries.
[Fe-L$_6$]: Metal-ligand environment involvivng two water molecules, Asp178, Asp267, His180, and the Lys backbone in water

O	0	-1.60689500	0.72141200	-1.95665800
O	0	-2.06396800	-0.30276800	1.19068800
C	0	2.40466400	1.66580800	0.99343800
O	0	1.25583600	1.28796700	1.26437500
C	-1	3.09179400	2.82703500	1.64650300
H	0	2.41198400	3.26981200	2.38098600
H	0	4.00087000	2.52856600	2.17481600
H	0	3.35788200	3.60843700	0.92945700
N	0	3.20458300	1.09314300	0.07609200
H	0	2.88611500	0.27643700	-0.43379200
C	0	-2.42807800	0.89443800	1.62071100
O	0	-0.01387900	2.29238200	-2.16227900
O	0	-2.81260500	1.08703400	2.77992700
C	0	-1.17260700	1.94819000	-1.87888200
C	-1	-2.34853800	1.99077600	0.53713900
C	-1	-2.16441200	3.03326300	-1.44135100
H	0	-3.21258200	2.78112300	-1.37477800
H	0	-1.80442300	3.69227200	-0.66468500
H	0	-2.13838700	3.70920600	-2.32865100
H	0	-2.86139800	2.88148400	0.89313100
H	0	-1.28295200	2.14953200	0.41604300
H	0	-2.83588600	1.50302000	-0.28050200
N	0	0.52817800	-1.73487200	-0.29681900
C	0	0.90565700	-2.13516400	0.91116900
C	0	1.64005100	-1.87809900	-1.11737000
H	0	0.28971300	-2.14081100	1.79794600
C	0	2.69969900	-2.36644200	-0.38890600
H	0	1.60562600	-1.62284900	-2.16686200
H	0	2.71408800	-2.87003200	1.69326000
N	0	2.19802600	-2.52511700	0.89234200
C	-1	4.11927200	-2.66853600	-0.74659900
H	0	4.36324300	-3.72197200	-0.56211900
H	0	4.28716600	-2.46228100	-1.80705900
H	0	4.81758400	-2.05674400	-0.16407800
C	-1	4.54101500	1.61455500	-0.10956800
H	0	5.02823000	1.02043400	-0.88840600
H	0	4.54198000	2.65202500	-0.45240900
H	0	5.15572600	1.52880200	0.79089100
Fe	0	-1.27950600	-0.73087600	-0.61218700
O	0	-2.82607800	-2.09195200	-1.20259000
H	0	-3.26862900	-2.40775400	-0.36569600
H	0	-3.52127200	-1.68273200	-1.74490700
O	0	-3.68971900	-2.49727400	1.31819900
H	0	-3.11540700	-1.72940500	1.56391400
H	0	-3.34025600	-3.26520500	1.79808200

Cartesian coordinates of optimized geometries.
[$Fe-L_6$]: Metal-ligand environment involvivng two water molecules, Asp178, Asp267, His180, and the Lys backbone in methanol

O	0	-1.60552300	0.71811500	-1.95601100
O	0	-2.05934200	-0.31603200	1.19315200
C	0	2.38804300	1.67179700	0.97447600
O	0	1.22512100	1.31872500	1.21470100
C	-1	3.09177400	2.80055800	1.66674200
H	0	2.40913500	3.24258700	2.39896300
H	0	3.98348200	2.46770000	2.20423300
H	0	3.38956400	3.59107000	0.97277900
N	0	3.19224600	1.09765700	0.06097500
H	0	2.86183900	0.30557900	-0.47926700
C	0	-2.43069800	0.87981200	1.62404600
O	0	-0.02118800	2.29275200	-2.19389100
O	0	-2.82351100	1.06769400	2.78064800
C	0	-1.17236500	1.94684500	-1.88626600
C	-1	-2.34776400	1.97862400	0.54287100
C	-1	-2.15809100	3.03029400	-1.43022700
H	0	-3.20670000	2.78154400	-1.36006900
H	0	-1.79012600	3.68033100	-0.64991400
H	0	-2.13490000	3.71483400	-2.31083900
H	0	-2.85822800	2.86982600	0.90069000
H	0	-1.28085000	2.13254300	0.42311700
H	0	-2.83476800	1.49390700	-0.27691200
N	0	0.53328900	-1.72014000	-0.28624900
C	0	0.92945100	-2.06478500	0.93273500
C	0	1.63175200	-1.90439900	-1.11699100
H	0	0.32850900	-2.02412300	1.82877000
C	0	2.70158400	-2.36216900	-0.38427200
H	0	1.58137100	-1.69637600	-2.17621100
H	0	2.74837000	-2.76834200	1.71896400
N	0	2.22022600	-2.46019500	0.91106400
C	-1	4.11486000	-2.68514800	-0.75075100
H	0	4.35833300	-3.73051800	-0.52581700
H	0	4.26705500	-2.52391200	-1.82125900
H	0	4.82394100	-2.05167700	-0.20504100
C	-1	4.54240200	1.59413200	-0.09233400
H	0	5.03031000	1.00437000	-0.87429500
H	0	4.57131600	2.63759100	-0.41533800
H	0	5.13957400	1.47810900	0.81621200
Fe	0	-1.27900000	-0.72779500	-0.61283600
O	0	-2.81037300	-2.11007900	-1.19997600
H	0	-3.25476000	-2.42302500	-0.36267200
H	0	-3.50647400	-1.71217300	-1.74943600
O	0	-3.68893600	-2.50543400	1.31455200
H	0	-3.11665900	-1.73698600	1.56416100

H	0	-3.35133400	-3.27072800	1.80679100

Cartesian coordinates of optimized geometries.
[$Mg-L_5$]: Metal-ligand environment involving one water molecule, Asp178, Asp267, His180, and the Lys backbone in gas phase.

O	0	-2.03546200	-0.44533200	-1.77047800
O	0	-1.96917100	-0.86060500	1.56858700
C	0	1.61490100	2.03412900	0.52001200
O	0	0.59137700	1.46141200	0.88142200
C	-1	2.01795500	3.43203700	0.91408300
H	0	1.25476100	3.82893200	1.58979900
H	0	2.97615200	3.46298000	1.43986200
H	0	2.07582800	4.09867500	0.04959900
N	0	2.55788500	1.47158800	-0.28370500
H	0	2.29255300	0.65067000	-0.81580400
C	0	-2.70595100	0.21261200	1.83323000
O	0	-0.96084000	1.46859500	-2.25688600
O	0	-3.08778000	0.52162200	2.95511600
C	0	-1.97386900	0.86531600	-1.90867500
C	-1	-3.04137700	1.02497100	0.54568700
C	-1	-3.24929200	1.65642100	-1.59761500
H	0	-4.16311300	1.11619600	-1.39843000
H	0	-3.09730900	2.53780400	-0.99328800
H	0	-3.45522600	2.11253200	-2.59700600
H	0	-3.79489800	1.77311800	0.78442000
H	0	-2.09012000	1.47364800	0.28373700
H	0	-3.39735200	0.25805500	-0.11050900
N	0	0.69801500	-1.82491000	0.09229500
C	0	1.22162700	-1.59028000	1.28644900
C	0	1.76245700	-2.02777900	-0.77168100
H	0	0.65543900	-1.34301000	2.17452600
C	0	2.94764400	-1.91597700	-0.09003700
H	0	1.60671000	-2.23269400	-1.82160400
H	0	3.19874200	-1.46727900	1.99268200
N	0	2.57161100	-1.64968400	1.22038600
C	-1	4.37217200	-1.98495100	-0.53428800
H	0	4.92382100	-2.78509000	-0.02434900
H	0	4.41637900	-2.18552400	-1.60852700
H	0	4.90001300	-1.04149400	-0.34558000
C	-1	3.64908500	2.30071500	-0.73323100
H	0	4.28605600	1.69964500	-1.39263300
H	0	3.32888600	3.17209800	-1.31572300
H	0	4.28089900	2.63407500	0.09283500
O	0	-2.41333100	-2.95678100	-0.87404100
H	0	-3.09682500	-3.29507600	-0.27290200
H	0	-2.87160100	-2.47170400	-1.58972700
Mg	0	-1.30940200	-1.29417300	-0.17813000

Cartesian coordinates of optimized geometries.
[Mg-L_5]: Metal-ligand environment involving one water molecule, Asp178, Asp267, His180, and the Lys backbone in water.

O	0	-2.18368900	-0.39149800	-1.66586400
O	0	-1.96422300	-0.69334800	1.59889300
C	0	2.00980300	2.19932200	0.83050600
O	0	1.43500000	1.55931800	1.72432200
C	-1	1.85230900	3.67320800	0.59323900
H	0	1.16034000	4.07752900	1.33839000
H	0	2.79741100	4.21325900	0.69275100
H	0	1.43908700	3.88861500	-0.39525400
N	0	2.86060300	1.66235000	-0.06112500
H	0	2.98128600	0.65711700	-0.08898500
C	0	-2.68377200	0.36322000	1.86321000
O	0	-1.21720100	1.54019600	-2.28165200
O	0	-2.96058400	0.72965400	3.01957900
C	0	-2.19041700	0.89287300	-1.85719800
C	-1	-3.15035100	1.12393200	0.59900200
C	-1	-3.48592900	1.65141500	-1.55621400
H	0	-4.36881300	1.08396800	-1.29885400
H	0	-3.36407400	2.57012300	-1.00059000
H	0	-3.74322400	2.04225300	-2.57053200
H	0	-3.92187700	1.84595100	0.86127100
H	0	-2.24428800	1.61149700	0.26224800
H	0	-3.50520800	0.32305900	-0.01322700
N	0	0.63729200	-1.92521600	0.07708000
C	0	1.22437100	-2.43663100	1.15256500
C	0	1.66209600	-1.59892700	-0.79970900
H	0	0.72499000	-2.79445300	2.04102900
C	0	2.87813600	-1.91124300	-0.24295500
H	0	1.46119100	-1.15647100	-1.76504600
H	0	3.23108700	-2.78542500	1.68156500
N	0	2.56605700	-2.44632200	0.99642500
C	-1	4.27839800	-1.74260900	-0.73611800
H	0	4.78227800	-2.70997100	-0.85196700
H	0	4.27233900	-1.24888900	-1.71160300
H	0	4.88146600	-1.13911500	-0.04708000
C	-1	3.42729000	2.50972700	-1.08600000
H	0	4.08412200	1.89365200	-1.70748100
H	0	2.67050700	2.94060600	-1.74784100
H	0	4.04290800	3.30933600	-0.66855200
O	0	-2.16916400	-3.20541700	-0.98240700
H	0	-2.28582700	-4.01292100	-0.45414600
H	0	-2.97178000	-3.11182100	-1.52367700
M	0	-1.41869200	-1.43254600	-0.16533300

Cartesian coordinates of optimized geometries.
[Mg-L_5]: Metal-ligand environment involving one water molecule, Asp178, Asp267, His180, and the Lys backbone in methanol.

O	0	-2.14623800	-0.38575600	-1.68775800
O	0	-1.96882300	-0.74014500	1.57311100
C	0	1.84302500	2.14894300	0.74590600
O	0	1.10694300	1.48590300	1.49017400
C	-1	1.89205700	3.64722700	0.70613900
H	0	1.17809100	4.04233400	1.43528200
H	0	2.87924400	4.03810200	0.96625200
H	0	1.61759800	4.04247500	-0.27516900
N	0	2.70657600	1.61958700	-0.13904000
H	0	2.70208900	0.61832100	-0.29549100
C	0	-2.68696500	0.31674500	1.84574700
O	0	-1.14639600	1.53683500	-2.27821400
O	0	-2.99371700	0.65331900	3.00287800
C	0	-2.13159300	0.90143000	-1.86519600
C	-1	-3.11708800	1.11297900	0.58884600
C	-1	-3.41652800	1.67758700	-1.56224000
H	0	-4.31221000	1.12192600	-1.32406400
H	0	-3.28331400	2.58391800	-0.98933700
H	0	-3.65598300	2.09135600	-2.57181000
H	0	-3.88301100	1.84042600	0.85185300
H	0	-2.19757500	1.59191600	0.27657000
H	0	-3.47021100	0.32818900	-0.04469600
N	0	0.66554500	-1.92691900	0.07159800
C	0	1.23922100	-2.21957500	1.23208500
C	0	1.70376600	-1.72282400	-0.82730300
H	0	0.72638200	-2.42472200	2.16028800
C	0	2.91456100	-1.89023100	-0.19813600
H	0	1.51584700	-1.46552700	-1.86012900
H	0	3.23927500	-2.40291200	1.85787200
N	0	2.58408900	-2.21142400	1.10914700
C	-1	4.32450000	-1.75261200	-0.67554700
H	0	4.88004900	-2.69163700	-0.56432200
H	0	4.33248200	-1.47858300	-1.73387100
H	0	4.86519200	-0.97929500	-0.11682600
C	-1	3.49054600	2.50752600	-0.96723100
H	0	4.12745500	1.89281600	-1.61070500
H	0	2.87598000	3.12946900	-1.62445300
H	0	4.15368200	3.14527900	-0.37859900
O	0	-2.15120400	-3.21428800	-1.00132300
H	0	-2.28210600	-4.01796000	-0.47068600
H	0	-2.94052300	-3.12178500	-1.56196100
Mg	0	-1.39161800	-1.44190900	-0.19560800

Cartesian coordinates of optimized geometries.
[Mg-L$_6$]: Metal-ligand environment involving two water molecules, Asp178, Asp267, His180, and the Lys backbone in gas phase.

O	0	-1.75258400	0.45768100	-1.55227900
O	0	-1.75390500	-2.09278300	0.67022200
C	0	0.63515000	1.37772600	1.40405700
O	0	-0.25472800	0.57939800	1.05067200
C	-1	0.72899100	1.99909700	2.77612300
H	0	-0.10765900	1.63986400	3.38220100
H	0	1.65239200	1.73197000	3.29582800
H	0	0.66474700	3.08896500	2.71964300
N	0	1.62142300	1.78920900	0.60184600
H	0	1.49995200	1.75339900	-0.41824700
C	0	-2.92923300	-1.70314400	1.12350300
O	0	-1.62201400	2.60454400	-0.99117700
O	0	-3.61077100	-2.33076000	1.93040000
C	0	-2.24268200	1.52256500	-1.00459300
C	-1	-3.38153800	-0.29311400	0.51827000
C	-1	-3.64658100	1.46116200	-0.36219000
H	0	-4.41882500	0.84587100	-0.78277700
H	0	-3.73205900	1.76127100	0.66981500
H	0	-3.99469600	2.45431000	-0.80492200
H	0	-4.36859400	-0.14601900	0.93868800
H	0	-2.60204700	0.30729800	0.95339700
H	0	-3.31877200	-0.53433800	-0.52432400
N	0	1.28703600	-1.55882900	-0.41125200
C	0	1.78870400	-2.29061400	0.56772600
C	0	2.35162800	-0.90052900	-0.99683300
H	0	1.21595600	-2.92313400	1.23269700
C	0	3.51596200	-1.22038700	-0.35247300
H	0	2.20374000	-0.20292100	-1.81039100
H	0	3.75158800	-2.57719700	1.29381100
N	0	3.13496100	-2.11646200	0.63748200
C	-1	4.89738400	-0.69166300	-0.51626600
H	0	5.63428900	-1.48590900	-0.69399300
H	0	4.93093000	-0.01435300	-1.37429700
H	0	5.22459100	-0.12764800	0.36719100
C	-1	2.64386000	2.62575300	1.16536700
H	0	3.37218000	2.84661300	0.37706500
H	0	2.27481400	3.59052800	1.53115300
H	0	3.19104100	2.12698200	1.96991500
Mg	0	-0.74923700	-0.91190700	-0.47414700
O	0	-0.85827100	-1.75408400	-2.49732400
H	0	-0.11154900	-2.06981800	-3.02583600
H	0	-1.23642700	-0.97168800	-2.94144600
O	0	0.82838900	2.10436400	-2.11095100
H	0	1.08998100	2.89781900	-2.60142000
H	0	-0.10746600	2.28558600	-1.79317100

Cartesian coordinates of optimized geometries.
[Mg-L$_6$]: Metal-ligand environment involving two water molecules, Asp178, Asp267, His180, and the Lys backbone in water

O	0	-1.78570000	0.51811000	-1.49975900
O	0	-1.78525000	-2.10530400	0.57156300
C	0	0.64747100	1.31914400	1.41840000
O	0	-0.26422200	0.53059200	1.05759200
C	-1	0.76291600	1.88643100	2.80407100
H	0	-0.07137300	1.52170700	3.41041700
H	0	1.68808300	1.58137000	3.29892000
H	0	0.72319900	2.97828000	2.79329300
N	0	1.61119800	1.75559200	0.61703900
H	0	1.51903900	1.67730200	-0.40049700
C	0	-2.95326200	-1.73804000	1.01439500
O	0	-1.70256700	2.68025900	-0.97109500
O	0	-3.66692600	-2.45147200	1.74802900
C	0	-2.28320700	1.57026700	-0.95335900
C	-1	-3.39415800	-0.29769600	0.52443700
C	-1	-3.67447800	1.49546800	-0.26852000
H	0	-4.46472000	0.90613100	-0.69523400
H	0	-3.72716500	1.73470400	0.78167100
H	0	-4.03002100	2.50797900	-0.64508000
H	0	-4.37672300	-0.16371100	0.95903800
H	0	-2.60435500	0.27256300	0.98277100
H	0	-3.33365400	-0.47023900	-0.53206900
N	0	1.30467100	-1.65732600	-0.34638600
C	0	1.87566600	-2.20409300	0.71844600
C	0	2.32548300	-1.05019800	-1.05863400
H	0	1.37187700	-2.75890800	1.49646000
C	0	3.51750900	-1.21106800	-0.40616100
H	0	2.13554600	-0.50224400	-1.97114500
H	0	3.86251700	-2.27920600	1.41902500
N	0	3.20714800	-1.96085400	0.71499300
C	-1	4.86221300	-0.65017200	-0.69117200
H	0	5.63342900	-1.42856000	-0.74571900
H	0	4.84626700	-0.12344900	-1.64917900
H	0	5.16825900	0.06452300	0.08291000
C	-1	2.64633300	2.58647600	1.18644500
H	0	3.36431900	2.81599400	0.39360800
H	0	2.26977100	3.54080700	1.56620600
H	0	3.19550200	2.07136400	1.97672000
Mg	0	-0.66430900	-0.86176700	-0.50553300
O	0	-0.83027200	-1.82926000	-2.41779300
H	0	-0.09625700	-2.32345300	-2.81690400
H	0	-1.15505400	-1.21248400	-3.09560400
O	0	0.80384600	2.29502900	-2.12413300
H	0	1.15967000	3.17674900	-2.31728800
H	0	-0.11376900	2.44935600	-1.77075600

Cartesian coordinates of optimized geometries.
[Mg-L_6]: Metal-ligand environment involving two water molecules, Asp178, Asp267, His180, and the Lys backbone in methanol

O	0	-1.78262800	0.51623500	-1.50012600
O	0	-1.78297500	-2.10465400	0.57543500
C	0	0.64580900	1.32112800	1.41863600
O	0	-0.26366200	0.53054500	1.05793600
C	-1	0.75805600	1.89306300	2.80290100
H	0	-0.07632100	1.52832800	3.40906900
H	0	1.68321000	1.59178100	3.30009500
H	0	0.71560200	2.98477300	2.78825900
N	0	1.61110400	1.75608600	0.61779000
H	0	1.51912700	1.67843800	-0.39977600
C	0	-2.95223600	-1.73860900	1.01691000
O	0	-1.70152400	2.67787900	-0.96973800
O	0	-3.66517100	-2.45073200	1.75162700
C	0	-2.28223100	1.56811500	-0.95448800
C	-1	-3.39438000	-0.29950600	0.52293400
C	-1	-3.67534500	1.49175100	-0.27403100
H	0	-4.46322500	0.90040600	-0.70228400
H	0	-3.73164200	1.73297900	0.77549100
H	0	-4.03127400	2.50317200	-0.65355300
H	0	-4.37816800	-0.16627200	0.95500700
H	0	-2.60656700	0.27267000	0.98229600
H	0	-3.33133800	-0.47406900	-0.53305100
N	0	1.30515500	-1.65544600	-0.34978600
C	0	1.87459600	-2.21207400	0.71052200
C	0	2.32692200	-1.04366900	-1.05626600
H	0	1.36926800	-2.77344300	1.48281900
C	0	3.51848300	-1.21086600	-0.40438100
H	0	2.13750800	-0.48740800	-1.96392500
H	0	3.86112400	-2.29654600	1.41113100
N	0	3.20648100	-1.96999200	0.71024600
C	-1	4.86376300	-0.64713500	-0.68228300
H	0	5.63550700	-1.42461400	-0.74206000
H	0	4.84952600	-0.11253800	-1.63595200
H	0	5.16815800	0.06138200	0.09816400
C	-1	2.64274900	2.59136200	1.18603600
H	0	3.36191500	2.81988100	0.39388100
H	0	2.26354800	3.54631400	1.56177200
H	0	3.19176500	2.08065100	1.97934100
Mg	0	-0.66561200	-0.86260400	-0.50273300
O	0	-0.82745000	-1.82225100	-2.42056000
H	0	-0.09783100	-2.31771600	-2.82575800
H	0	-1.15717300	-1.20337200	-3.09400600
O	0	0.80422700	2.28985100	-2.12229400
H	0	1.15604800	3.17027300	-2.32804500
H	0	-0.11426200	2.44529500	-1.77104900

Cartesian coordinates of optimized geometries.
[Co-L_5]: Metal-ligand environment involving one water molecule, Asp178, Asp267, His180, and the Lys backbone in gas phase.

O	0	-2.05998100	-0.26424400	-1.68900600
O	0	-1.90266000	-0.57413800	1.54435200
C	0	1.96862300	2.01161400	0.53903400
O	0	0.98358500	1.49086400	1.05246800
C	-1	2.38233200	3.44631700	0.71912600
H	0	1.67252100	3.92271600	1.40163900
H	0	3.38057000	3.54836500	1.15366500
H	0	2.35809900	3.99886700	-0.22403100
N	0	2.84982600	1.35335000	-0.26320900
H	0	2.54780300	0.47741700	-0.67455100
C	0	-2.52322300	0.57357500	1.83002900
O	0	-0.77367700	1.51507800	-2.18733600
O	0	-2.81649400	0.90161800	2.97076600
C	0	-1.85362900	1.03330600	-1.85883100
C	-1	-2.84588900	1.40515900	0.55841500
C	-1	-3.05310300	1.94899100	-1.60863400
H	0	-4.01158600	1.49849600	-1.39210200
H	0	-2.83443900	2.84157600	-1.04224300
H	0	-3.21244200	2.37170700	-2.63114800
H	0	-3.51342200	2.22304800	0.82664500
H	0	-1.87169600	1.76125200	0.24825900
H	0	-3.30545400	0.66554500	-0.06091000
N	0	0.65620100	-1.68347600	0.12643700
C	0	1.20375600	-1.49082000	1.31683400
C	0	1.69272400	-1.99292700	-0.74203200
H	0	0.66755400	-1.18230800	2.20299800
C	0	2.88747900	-1.98882400	-0.06444100
H	0	1.51085900	-2.18780000	-1.78938000
H	0	3.18594500	-1.54127500	2.01253900
N	0	2.54127800	-1.68284700	1.24624800
C	-1	4.30356600	-2.19168300	-0.50777400
H	0	4.77448200	-3.04164000	0.00227300
H	0	4.33072300	-2.39347400	-1.58234300
H	0	4.91684200	-1.30240500	-0.31486000
C	-1	3.89078600	2.12150700	-0.90057000
H	0	4.47954200	1.44355400	-1.52960400
H	0	3.51413200	2.91286100	-1.55870100
H	0	4.58379900	2.55569700	-0.17683600
O	0	-2.56100800	-2.76339800	-0.75339000
H	0	-3.21648500	-2.95063200	-0.06059500
H	0	-3.02907900	-2.26739500	-1.45320700
Co	0	-1.27918800	-1.14692200	-0.15976400

Cartesian coordinates of optimized geometries.
[Co-L_5]: Metal-ligand environment involving one water molecule, Asp178, Asp267, His180, and the Lys backbone in water.

O	0	-2.03260600	-0.16818200	-1.66421700
O	0	-1.87017000	-0.63713000	1.57365200
C	0	1.78312300	2.15114600	0.50930400
O	0	0.69489400	1.65783100	0.83565200
C	-1	2.33210500	3.43193700	1.06240500
H	0	1.61217300	3.84232200	1.77716700
H	0	3.27676200	3.28361600	1.59214400
H	0	2.49374400	4.18494100	0.28661500
N	0	2.63291500	1.61467000	-0.38604700
H	0	2.42398900	0.71614200	-0.80661700
C	0	-2.52691700	0.46235100	1.85332000
O	0	-0.89752600	1.59408700	-2.46684100
O	0	-2.85108600	0.76663900	3.01402600
C	0	-1.88873400	1.10944900	-1.90235800
C	-1	-2.85469900	1.32825900	0.61735300
C	-1	-3.02998800	2.05506400	-1.49825200
H	0	-3.99081200	1.63335700	-1.24770900
H	0	-2.72987300	2.89374200	-0.88642300
H	0	-3.23610800	2.55839200	-2.47186800
H	0	-3.52956700	2.13384600	0.90001700
H	0	-1.87619500	1.68892300	0.32815600
H	0	-3.29048400	0.60551000	-0.04230800
N	0	0.72075600	-1.71023900	0.09517300
C	0	1.27024600	-1.76983300	1.30149600
C	0	1.76407500	-1.82001000	-0.81590500
H	0	0.74232800	-1.69810000	2.24045100
C	0	2.95808900	-1.94133100	-0.14461100
H	0	1.58849000	-1.80336500	-1.88196300
H	0	3.25037800	-1.97834400	1.97701200
N	0	2.60876800	-1.91139200	1.19579800
C	-1	4.37701300	-2.04641600	-0.60823000
H	0	4.84753900	-2.97035400	-0.25174600
H	0	4.41191800	-2.04620300	-1.70086100
H	0	4.97623800	-1.20273000	-0.24641900
C	-1	3.89490100	2.27740900	-0.63449600
H	0	4.44101000	1.69135300	-1.37999300
H	0	3.76517500	3.27942300	-1.04988300
H	0	4.52669600	2.33213600	0.25642500
O	0	-2.35937700	-2.91356700	-0.73687500
H	0	-2.66612400	-3.47963300	-0.00751000
H	0	-3.15718500	-2.66187800	-1.23459800
Co	0	-1.24316600	-1.21407200	-0.19881100

Cartesian coordinates of optimized geometries.
[Co-L_5]: Metal-ligand environment involving one water molecule, Asp178, Asp267, His180, and the Lys backbone in methanol.

O	0	-2.03182700	-0.17483000	-1.66373800
O	0	-1.87248700	-0.63133100	1.57524400
C	0	1.79039300	2.13801900	0.49753300
O	0	0.69864800	1.65331700	0.82376400
C	-1	2.34682500	3.41834600	1.04493700
H	0	1.62877800	3.83622400	1.75718800
H	0	3.29034200	3.26765300	1.57606200
H	0	2.51284600	4.16650800	0.26537300
N	0	2.64018100	1.59098000	-0.39196600
H	0	2.41887200	0.69986100	-0.82161300
C	0	-2.52695000	0.47125900	1.85174100
O	0	-0.89046600	1.58049500	-2.47309400
O	0	-2.85404800	0.77775400	3.01035400
C	0	-1.88316800	1.10186500	-1.90724100
C	-1	-2.84926100	1.33504000	0.61254200
C	-1	-3.02199100	2.05135900	-1.50686100
H	0	-3.98410300	1.63273300	-1.25595200
H	0	-2.72036300	2.89147900	-0.89790700
H	0	-3.22607400	2.55201700	-2.48233400
H	0	-3.52130100	2.14412200	0.89204000
H	0	-1.86913900	1.69098600	0.32283900
H	0	-3.28761500	0.61194900	-0.04487400
N	0	0.71591000	-1.69865500	0.10426200
C	0	1.27006100	-1.69959600	1.30983300
C	0	1.75497900	-1.85539100	-0.80472500
H	0	0.74513100	-1.57740400	2.24516500
C	0	2.95117000	-1.94672300	-0.13303200
H	0	1.57518400	-1.89015900	-1.86959700
H	0	3.25257100	-1.87888500	1.98660600
N	0	2.60759900	-1.85020700	1.20588900
C	-1	4.36733100	-2.07757800	-0.59712900
H	0	4.83834900	-2.98345500	-0.19739000
H	0	4.39709100	-2.13239300	-1.68856900
H	0	4.97035200	-1.21821600	-0.28132900
C	-1	3.90400700	2.24809300	-0.64618500
H	0	4.44817000	1.65476400	-1.38739400
H	0	3.77790800	3.24760000	-1.06906300
H	0	4.53604800	2.30734100	0.24419500
O	0	-2.37860100	-2.90713200	-0.72139000
H	0	-2.71584800	-3.44704300	0.01422400
H	0	-3.15862800	-2.64623900	-1.24212300
Co	0	-1.24609300	-1.21180000	-0.19372000

Cartesian coordinates of optimized geometries.
[Co-L$_6$]: Metal-ligand environment involving two water molecules, Asp178, Asp267, His180, and the Lys backbone in gas phase

O	0	-1.75008700	-1.22559700	-1.43115300
O	0	-1.10636700	-1.23567200	1.75436800
C	0	0.08670600	3.79670400	0.54299800
O	0	-0.91906400	4.44909000	0.32955400
C	-1	0.47684700	3.16396200	1.84837000
H	0	-0.34683500	3.30225600	2.55479600
H	0	0.65415400	2.08883700	1.75960000
H	0	1.36402900	3.62878800	2.28569200
N	0	1.02970500	3.49778900	-0.42536500
H	0	0.97618400	4.06239000	-1.26898600
C	0	-2.40103000	-1.31254400	2.08614000
O	0	-2.07423000	0.82277300	-2.27964300
O	0	-2.78054700	-1.66401500	3.19202800
C	0	-2.49088000	-0.17255700	-1.65402900
C	-1	-3.31976500	-0.90743600	0.90644200
C	-1	-3.90636300	-0.12928800	-1.11469000
H	0	-4.40166400	-1.04703200	-0.85937600
H	0	-4.12237500	0.71375300	-0.47204100
H	0	-4.45130900	0.18321100	-2.03783400
H	0	-4.35957500	-0.87533900	1.22911100
H	0	-2.93258500	0.06994200	0.68033600
H	0	-3.11164200	-1.66979700	0.17600400
N	0	1.44379800	-1.63104700	0.21007100
C	0	1.99182100	-1.96633600	1.36812500
C	0	2.47364500	-1.28131000	-0.64466900
H	0	1.44647700	-2.26587100	2.25226900
C	0	3.66717900	-1.38371400	0.01341000
H	0	2.26629800	-0.98772800	-1.66374200
H	0	3.99103300	-2.03555800	2.03511000
N	0	3.33895100	-1.83105700	1.28941400
C	-1	5.04617200	-1.03260000	-0.41688800
H	0	5.73947100	-1.88042600	-0.33874000
H	0	5.03196100	-0.71605700	-1.46348000
H	0	5.46262900	-0.20794600	0.17676100
C	-1	2.35079400	3.15610300	0.07583600
H	0	3.02076300	3.05161700	-0.78470400
H	0	2.77906800	3.92373900	0.73073600
H	0	2.35695700	2.19595600	0.59041500
O	0	0.39241700	-1.76690600	-3.30575300
H	0	0.20152500	-1.38810500	-4.17648700
H	0	-0.42826000	-1.63165400	-2.78711600
O	0	0.19326000	0.78943600	-1.04213900
H	0	0.45863900	1.69830900	-0.78552700
H	0	-0.54292200	0.90690100	-1.71900300
Co	0	-0.41603800	-0.90032400	0.02085200

Cartesian coordinates of optimized geometries.
[Co-L$_6$]: Metal-ligand environment involving two water molecules, Asp178, Asp267, His180, and the Lys backbone in water

O	0	1.83305500	-1.21869900	1.21337100
O	0	1.02193000	-1.11149500	-1.93899100
C	0	-0.10714800	3.73480600	-0.24347800
O	0	1.02673200	4.12502000	0.03522500
C	-1	-0.63262300	3.49712600	-1.62696100
H	0	0.17861900	3.66183000	-2.34239100
H	0	-0.99567000	2.47609400	-1.76580600
H	0	-1.44150500	4.18656100	-1.88015800
N	0	-1.05286500	3.42114600	0.69375700
H	0	-0.89734500	3.77716600	1.63322200
C	0	2.28078100	-1.01645300	-2.31028300
O	0	2.10731600	0.73704800	2.27304700
O	0	2.65009400	-1.24908200	-3.47046600
C	0	2.52722600	-0.15689600	1.50574200
C	-1	3.22462600	-0.59983600	-1.16381700
C	-1	3.89517100	0.02798500	0.88326000
H	0	4.43065800	-0.83391200	0.53002100
H	0	4.01259500	0.92485300	0.29035300
H	0	4.48422900	0.31005700	1.78799100
H	0	4.24817200	-0.50262200	-1.52257400
H	0	2.80792300	0.35057000	-0.87996200
H	0	3.08251800	-1.39990200	-0.46081200
N	0	-1.53125000	-1.64379500	-0.24859100
C	0	-2.15923300	-2.15632300	-1.30124700
C	0	-2.51252000	-1.15275700	0.59671800
H	0	-1.69148900	-2.61710400	-2.15827300
C	0	-3.74188900	-1.34895900	0.03878300
H	0	-2.26436900	-0.65980000	1.52241900
H	0	-4.19086700	-2.30245600	-1.82998500
N	0	-3.49416100	-1.99915800	-1.15990800
C	-1	-5.07410800	-0.89996000	0.50842500
H	0	-5.78477800	-1.73088400	0.59942800
H	0	-4.97598900	-0.43067000	1.49082500
H	0	-5.51412000	-0.16437700	-0.17647600
C	-1	-2.42662100	3.33801100	0.21959100
H	0	-3.06758500	3.15674900	1.08753800
H	0	-2.77029200	4.26280500	-0.25367300
H	0	-2.57283500	2.50099300	-0.46095600
O	0	0.82352500	-2.76310500	3.37115500
H	0	0.20922300	-2.21238300	3.88118400
H	0	1.13890400	-2.18365800	2.64333100
O	0	-0.26137700	0.64287900	1.16940400
H	0	-0.60810600	1.52898500	0.92614500
H	0	0.51550500	0.80298400	1.77587500
Co	0	0.33194000	-0.89815600	-0.12209000

Cartesian coordinates of optimized geometries.
[Co-L_6]: Metal-ligand environment involving two water molecules, Asp178, Asp267, His180, and the Lys backbone in methanol

O	0	1.83710000	-1.21813500	1.20771700
O	0	1.01997700	-1.11062700	-1.94133100
C	0	-0.12399900	3.74053700	-0.24687500
O	0	1.00593000	4.14223000	0.02885700
C	-1	-0.64919800	3.49191200	-1.62854600
H	0	0.16054500	3.65723600	-2.34547300
H	0	-1.00731400	2.46849500	-1.76216700
H	0	-1.46183500	4.17580700	-1.88488700
N	0	-1.06618100	3.42122200	0.69319600
H	0	-0.91246000	3.78382300	1.63047300
C	0	2.27848100	-1.01246800	-2.31615500
O	0	2.10677400	0.73527900	2.27197800
O	0	2.64572400	-1.24555000	-3.47603100
C	0	2.52849800	-0.15454200	1.50097000
C	-1	3.22221000	-0.59226300	-1.17067100
C	-1	3.89379700	0.03801900	0.87532000
H	0	4.43313200	-0.82084300	0.52056300
H	0	4.00426800	0.93548200	0.28205600
H	0	4.48334400	0.32359800	1.77848400
H	0	4.24359000	-0.48343600	-1.53205300
H	0	2.79604600	0.35228800	-0.88130100
H	0	3.08976100	-1.39673300	-0.47061000
N	0	-1.52653100	-1.64321600	-0.24608300
C	0	-2.15341700	-2.17236200	-1.29098200
C	0	-2.50893900	-1.14972800	0.59640300
H	0	-1.68432000	-2.63952800	-2.14381500
C	0	-3.73844800	-1.36184300	0.04461200
H	0	-2.26153300	-0.64618200	1.51657600
H	0	-4.18552100	-2.34036900	-1.81172300
N	0	-3.48921300	-2.02350400	-1.14757000
C	-1	-5.07298700	-0.91945300	0.51416900
H	0	-5.77596100	-1.75554500	0.61671400
H	0	-4.97560700	-0.43924800	1.49136400
H	0	-5.52211600	-0.19487200	-0.17654800
C	-1	-2.43989700	3.32717200	0.22073800
H	0	-3.07907000	3.14797300	1.09048800
H	0	-2.78984300	4.24648500	-0.25849800
H	0	-2.58185100	2.48436700	-0.45366500
O	0	0.83003300	-2.74018200	3.38602100
H	0	0.50227100	-2.12094200	4.05672900
H	0	1.15295200	-2.17191300	2.65279700
O	0	-0.25585100	0.64850600	1.15768800
H	0	-0.60334500	1.53603700	0.92001000
H	0	0.51801300	0.80523600	1.76938400
Co	0	0.33689700	-0.89965900	-0.12499200

Cartesian coordinates of optimized geometries.
[Ni-L_5]: Metal-ligand environment involving one molecule of water, Asp178, Asp267, His180, and the Lys backbone in gas phase

O	0	-3.04345800	-0.32358700	-1.53012600
O	0	-1.77640000	-0.29102600	1.34803100
C	0	2.62938800	1.99115500	0.66141000
O	0	2.02072900	1.55947800	1.63443600
C	-1	2.63033600	3.42086100	0.19647200
H	0	2.02390900	4.00776100	0.89249600
H	0	3.63164300	3.86033200	0.17949700
H	0	2.19104100	3.52417800	-0.79907200
N	0	3.39766600	1.21466200	-0.15613300
H	0	3.17919100	0.22568000	-0.15838500
C	0	-2.34259800	0.85141200	1.74062100
O	0	-1.08298100	0.66576200	-1.73305200
O	0	-2.56974000	1.10000500	2.91708000
C	0	-2.35543700	0.74234100	-1.69458300
C	-1	-2.72334600	1.76510200	0.54809900
C	-1	-3.02709200	2.08610000	-1.65188700
H	0	-4.04985300	2.09787800	-1.30058500
H	0	-2.42823900	2.91518000	-1.30683800
H	0	-3.15198800	2.32865600	-2.73988100
H	0	-3.11310400	2.71908300	0.91196800
H	0	-1.78514500	1.89054400	0.04425900
H	0	-3.50089500	1.17821400	0.10425100
N	0	0.49555300	-1.55604600	-0.02362100
C	0	0.99542500	-1.42923800	1.19479700
C	0	1.53599600	-1.95911400	-0.84147200
H	0	0.44698500	-1.06536800	2.05181900
C	0	2.68948800	-2.08419400	-0.10685100
H	0	1.39398600	-2.12254400	-1.90045100
H	0	2.92492800	-1.64946600	1.98622000
N	0	2.30901700	-1.75732400	1.19066200
C	-1	4.08825000	-2.44275200	-0.49444200
H	0	4.41988700	-3.37458500	-0.01797700
H	0	4.14558400	-2.58717800	-1.57719200
H	0	4.80714600	-1.65967400	-0.22093200
C	-1	3.94322200	1.82192600	-1.34421000
H	0	4.51632800	1.06035200	-1.88613100
H	0	3.18455900	2.20230400	-2.03927100
H	0	4.64514000	2.62194500	-1.10449400
O	0	-2.38496300	-2.65239600	0.43154800
H	0	-2.46997400	-2.09974600	1.24527400
H	0	-3.27929200	-2.69683400	0.05291000
Ni	0	-1.38695600	-0.99047200	-0.47446100

Cartesian coordinates of optimized geometries.
[Ni-L_5]: Metal-ligand environment involving one molecule of water, Asp178, Asp267, His180, and the Lys backbone in water

O	0	-3.35536900	0.11086300	-1.37036200
O	0	-1.93051800	0.09245200	1.44133400
C	0	4.10996900	1.98925100	0.44796700
O	0	4.56062400	2.18759900	1.58674700
C	-1	3.02342300	2.80439200	-0.20092100
H	0	2.69871800	3.57692100	0.50275500
H	0	3.37195700	3.30680200	-1.10708100
H	0	2.14998700	2.20112600	-0.45901000
N	0	4.53108500	1.01326400	-0.37554400
H	0	5.26535900	0.38044200	-0.08156400
C	0	-2.20004300	1.33647900	1.74580900
O	0	-1.25383700	0.68197000	-1.70821200
O	0	-2.21936400	1.74647800	2.92011200
C	0	-2.48596200	1.00740700	-1.63825500
C	-1	-2.51458000	2.22146200	0.51915000
C	-1	-2.88336500	2.45590800	-1.68182300
H	0	-3.85490000	2.69886300	-1.27327600
H	0	-2.11307500	3.17611200	-1.44739300
H	0	-3.04078200	2.63585800	-2.77632900
H	0	-2.70492100	3.25471400	0.81888000
H	0	-1.60700000	2.14642000	-0.04669800
H	0	-3.41161400	1.75526400	0.16714700
N	0	0.00956200	-1.66813000	0.09860200
C	0	0.58154500	-1.66665900	1.29460300
C	0	0.91195200	-2.25765700	-0.77193400
H	0	0.14632200	-1.26576300	2.19701300
C	0	2.04809500	-2.61596900	-0.08957600
H	0	0.69034100	-2.37972900	-1.82242300
H	0	2.44594100	-2.34417000	1.99722600
N	0	1.80793000	-2.23073800	1.21877400
C	-1	3.31901100	-3.26094400	-0.53049800
H	0	3.50132100	-4.20047500	0.00526500
H	0	3.26964800	-3.48434200	-1.59962600
H	0	4.18208300	-2.60599900	-0.35975500
C	-1	3.92123400	0.89167100	-1.68060500
H	0	4.40166500	0.05676100	-2.19946300
H	0	2.85451400	0.66138700	-1.62468200
H	0	4.07143800	1.78052000	-2.29823500
O	0	-2.92384900	-2.33350700	0.65930800
H	0	-3.05736600	-1.85739400	1.50206300
H	0	-3.81266700	-2.45341600	0.28167400
Ni	0	-1.78200600	-0.86284100	-0.34586600

Cartesian coordinates of optimized geometries.
[Ni-L_5]: Metal-ligand environment involving one molecule of water, Asp178, Asp267, His180, and the Lys backbone in methanol

O	0	-3.33808300	0.10759100	-1.38024100
O	0	-1.92415300	0.07151100	1.43499200
C	0	4.06943200	2.00322600	0.48773400
O	0	4.49126800	2.17023900	1.64208300
C	-1	3.01071000	2.84651100	-0.17237800
H	0	2.67033600	3.60185200	0.54231700
H	0	3.39368100	3.37082100	-1.05184600
H	0	2.14079800	2.25934000	-0.47575700
N	0	4.50212500	1.04070100	-0.34666700
H	0	5.22003800	0.39154400	-0.04696200
C	0	-2.21253900	1.30872200	1.75372800
O	0	-1.24218900	0.70433800	-1.70722900
O	0	-2.25326800	1.70079700	2.93273100
C	0	-2.47731300	1.01647100	-1.63589400
C	-1	-2.52263100	2.20737600	0.53545300
C	-1	-2.89118100	2.46072100	-1.66345400
H	0	-3.86979400	2.68492500	-1.26061300
H	0	-2.13261800	3.18729600	-1.41214000
H	0	-3.04101000	2.65351800	-2.75686100
H	0	-2.71889600	3.23588800	0.84738900
H	0	-1.61197000	2.14314000	-0.02709900
H	0	-3.41633300	1.74320600	0.17338500
N	0	0.02237500	-1.68204700	0.07334100
C	0	0.55118100	-1.77653300	1.28528600
C	0	0.95934500	-2.19544700	-0.80915600
H	0	0.08373700	-1.44929300	2.20130300
C	0	2.07193700	-2.60572400	-0.11726400
H	0	0.77706100	-2.23040200	-1.87360300
H	0	2.39175100	-2.50493000	1.99843100
N	0	1.78253200	-2.32913000	1.20856800
C	-1	3.36047300	-3.21243900	-0.56294000
H	0	3.50438400	-4.21290500	-0.13781400
H	0	3.36981700	-3.30306600	-1.65252900
H	0	4.21712500	-2.59570900	-0.26518400
C	-1	3.92702200	0.95687900	-1.67028100
H	0	4.41902700	0.13476400	-2.19916900
H	0	2.85825600	0.72945100	-1.64976000
H	0	4.09697900	1.86136100	-2.25921600
O	0	-2.90601600	-2.32789900	0.64956000
H	0	-3.03407000	-1.82503000	1.47849800
H	0	-3.79602700	-2.45543800	0.27758700
Ni	0	-1.76323500	-0.86065000	-0.36275800

Cartesian coordinates of optimized geometries.
[Ni-L_6]: Metal-ligand environment involving two molecules of water, Asp178, Asp267, His180, and the Lys backbone in gas phase.

O	0	-2.36962500	-1.17488000	-1.13000800
O	0	-1.07923100	-0.89938100	1.66726900
C	0	0.44077800	3.45154800	0.06385300
O	0	-0.74376800	3.55448600	-0.21027600
C	-1	1.05763700	3.74018200	1.40526000
H	0	0.25172200	3.98680000	2.10224300
H	0	1.59203300	2.87797800	1.81339000
H	0	1.74203100	4.59158100	1.37295400
N	0	1.39862600	3.02928800	-0.83512700
H	0	1.14334600	3.12132100	-1.81436600
C	0	-2.21508300	-0.47507000	2.23379800
O	0	-2.05821800	0.68470100	-2.34549400
O	0	-2.38791100	-0.52794000	3.44553100
C	0	-2.69472300	0.02491000	-1.48972400
C	-1	-3.24026400	0.08377200	1.21335600
C	-1	-3.88632100	0.68818500	-0.84866800
H	0	-4.62110600	0.10040700	-0.33121900
H	0	-3.68181900	1.65902500	-0.41477700
H	0	-4.44364500	1.00386500	-1.76661300
H	0	-4.11452600	0.48836700	1.72464100
H	0	-2.65300200	0.85231400	0.74971500
H	0	-3.44730100	-0.78592600	0.61870000
N	0	1.38276200	-1.65323800	0.39625000
C	0	1.97609700	-1.52152800	1.56747800
C	0	2.37760500	-1.60659900	-0.55966900
H	0	1.46489500	-1.49384600	2.51991100
C	0	3.59568600	-1.42548300	0.02934400
H	0	2.15502100	-1.64885200	-1.61342400
H	0	3.99816900	-1.28554800	2.13701900
N	0	3.32057300	-1.38318600	1.39252400
C	-1	4.93129700	-1.21958800	-0.59903600
H	0	5.66787800	-1.96883200	-0.28019200
H	0	4.83373400	-1.29452600	-1.68560500
H	0	5.34620300	-0.22944400	-0.36841800
C	-1	2.77788300	3.29684100	-0.46500600
H	0	3.41966100	2.97572000	-1.29346400
H	0	2.98778000	4.35825200	-0.28777800
H	0	3.07954500	2.71756100	0.40693100
Ni	0	-0.56400200	-1.32085500	-0.13041200
O	0	-0.50387700	-2.85758200	-1.72580300
H	0	0.10758700	-2.67319000	-2.45584900
H	0	-1.37272800	-2.48510000	-2.00515600
O	0	0.24767100	0.05967300	-1.49729800
H	0	0.63689500	0.85382400	-1.08493500
H	0	-0.57792900	0.37651700	-2.00498400

Cartesian coordinates of optimized geometries.
[Ni-L_6]: Metal-ligand environment involving two molecules of water, Asp178, Asp267, His180, and the Lys backbone in water

O	0	-2.18751600	-1.03991400	-0.99974800
O	0	-1.04675300	-0.81342200	1.97326000
C	0	0.67366600	3.49472800	-0.12641000
O	0	-0.47752300	3.85774100	-0.37616500
C	-1	1.32321000	3.53870100	1.22455300
H	0	0.58355100	3.87303400	1.95815100
H	0	1.68815700	2.55892400	1.54152900
H	0	2.15843500	4.24252800	1.25248600
N	0	1.52174900	2.96844300	-1.05755100
H	0	1.28495900	3.12985100	-2.03259500
C	0	-2.20235900	-0.39743100	2.44346300
O	0	-2.03563400	0.86074500	-2.17795600
O	0	-2.46668800	-0.43203400	3.65589400
C	0	-2.61870400	0.13986100	-1.33733000
C	-1	-3.16871400	0.12084300	1.36126700
C	-1	-3.87326900	0.68302700	-0.69352000
H	0	-4.55760500	0.00381300	-0.21809200
H	0	-3.76076100	1.63997000	-0.20127500
H	0	-4.44550300	0.99000400	-1.60183700
H	0	-4.08371500	0.51007200	1.80586600
H	0	-2.57322000	0.89470800	0.91114100
H	0	-3.31577900	-0.75907600	0.76288300
N	0	1.36208600	-1.67908600	0.55956700
C	0	2.07974300	-1.48062400	1.65713900
C	0	2.26409600	-1.84004400	-0.47928400
H	0	1.68609500	-1.30891500	2.64738200
C	0	3.54065400	-1.71590600	-0.00958900
H	0	1.93719600	-1.99087900	-1.49651900
H	0	4.15132400	-1.37795400	2.01967500
N	0	3.39817400	-1.50121200	1.35325100
C	-1	4.83173700	-1.69859400	-0.74588200
H	0	5.53547400	-2.45037200	-0.36774700
H	0	4.65092500	-1.90663200	-1.80378900
H	0	5.32169600	-0.71990600	-0.67108000
C	-1	2.93162700	2.93179700	-0.69855900
H	0	3.48629800	2.55762100	-1.56439100
H	0	3.33523200	3.91926300	-0.45359200
H	0	3.12329300	2.24196400	0.12150700
Ni	0	-0.50505800	-1.12023700	0.10923400
O	0	-1.82968800	-2.94241200	-3.08205600
H	0	-1.30554100	-2.52973000	-3.78616600
H	0	-1.92260900	-2.24790400	-2.39390600
O	0	0.30945700	0.18675200	-1.27234800
H	0	0.83600400	0.96934800	-1.00817100
H	0	-0.46172100	0.53207100	-1.80902300

Cartesian coordinates of optimized geometries.

[Ni-L_6]: Metal-ligand environment involving two molecules of water, Asp178, Asp267, His180, and the Lys backbone in methanol

O	0	-2.17535900	-1.05791800	-1.01502800
O	0	-1.03191000	-0.86073500	1.95965900
C	0	0.62676500	3.53231500	-0.08093100
O	0	-0.52462700	3.89385100	-0.32821000
C	-1	1.27302800	3.55428900	1.27195800
H	0	0.53004000	3.87194000	2.00957000
H	0	1.64150400	2.57068000	1.57257400
H	0	2.10500700	4.26120400	1.31521400
N	0	1.47975900	3.02275700	-1.01805300
H	0	1.24260700	3.19557500	-1.99110500
C	0	-2.18707100	-0.45595900	2.44249800
O	0	-2.05160200	0.86366900	-2.16273100
O	0	-2.44157100	-0.50259800	3.65612400
C	0	-2.62255700	0.12214200	-1.33196900
C	-1	-3.16555900	0.06624500	1.37229200
C	-1	-3.87990700	0.64035100	-0.67584000
H	0	-4.55480600	-0.05260200	-0.20688400
H	0	-3.77756600	1.59318300	-0.17381500
H	0	-4.45918500	0.95129700	-1.57857700
H	0	-4.08456100	0.43514000	1.82589000
H	0	-2.58363000	0.85608800	0.93202200
H	0	-3.30175300	-0.80659100	0.76132800
N	0	1.37947200	-1.68840500	0.51230200
C	0	2.08603500	-1.59619700	1.63054800
C	0	2.29131100	-1.75589100	-0.52815500
H	0	1.68218500	-1.51549900	2.62826700
C	0	3.56332000	-1.68176600	-0.03663400
H	0	1.97437100	-1.81113900	-1.55798700
H	0	4.15365500	-1.53500900	2.02206700
N	0	3.40770200	-1.59364800	1.33898300
C	-1	4.86080400	-1.60601700	-0.75801400
H	0	5.55390900	-2.39867600	-0.45017500
H	0	4.68660000	-1.70810300	-1.83238200
H	0	5.36014100	-0.64472500	-0.58465600
C	-1	2.88959400	2.99372400	-0.65840100
H	0	3.44824200	2.63475000	-1.52812700
H	0	3.28443000	3.98171400	-0.40131500
H	0	3.08714600	2.29638400	0.15383100
Ni	0	-0.49350000	-1.13439400	0.08846900
O	0	-1.74321300	-2.90152900	-3.14196500
H	0	-1.48932600	-2.40065800	-3.93280800
H	0	-1.87619600	-2.22424800	-2.44354800
O	0	0.29116800	0.25483300	-1.22020100
H	0	0.80679400	1.04163400	-0.94827200
H	0	-0.47781800	0.58977400	-1.76617700
